# Functional Characterization of *Phalaenopsis aphrodite* Flowering Genes PaFT1 and PaFD

**DOI:** 10.1371/journal.pone.0134987

**Published:** 2015-08-28

**Authors:** Seonghoe Jang, Sang-Chul Choi, Hsing-Yi Li, Gynheung An, Elmon Schmelzer

**Affiliations:** 1 Biotechnology Center in Southern Taiwan, Academia Sinica, Tainan County, 741, Taiwan; 2 Agricultural Biotechnology Research Center, Academia Sinica, Taipei, 115, Taiwan; 3 Crop Biotechnology Center, Kyunghee University, Yongin, 446–701, Korea; 4 Max-Planck-Institute for Plant breeding research, Cologne, 50829, Germany; Ecole Normale Superieure, FRANCE

## Abstract

We show that the key flowering regulators encoded by *Phalaenopsis aphrodite FLOWERING LOCUS T1* (*PaFT1*) and *PaFD* share high sequence homologies to these from long-day flowering *Arabidopsis* and short-day flowering rice. Interestingly, *PaFT1* is specifically up-regulated during flowering inductive cooling treatment but is not subjected to control by photoperiod in *P*. *aphrodite*. Phloem or shoot apex-specific expression of *PaFT1* restores the late flowering of *Arabidopsis ft* mutants. Moreover, *PaFT1* can suppress the delayed flowering caused by *SHORT VEGATATIVE PHASE* (*SVP*) overexpression as well as an active *FRIGIDA* (*FRI*) allele, indicating the functional conservation of flowering regulatory circuit in different plant species. *PaFT1* promoter:*GUS* in *Arabidopsis* showed similar staining pattern to that of *Arabidopsis FT* in the leaves and guard cells but different in the shoot apex. A genomic clone or heat shock-inducible expression of *PaFT1* is sufficient to the partial complementation of the *ft* mutants. Remarkably, ectopic *PaFT1* expression also triggers precocious heading in rice. To further demonstrate the functional conservation of the flowering regulators, we show that PaFD, a bZIP transcription factor involved in flowering promotion, interacts with PaFT1, and *PaFD* partially complemented *Arabidopsis fd* mutants. Transgenic rice expressing *PaFD* also flowered early with increased expression of rice homologues of *APETALA1* (*AP1*). Consistently, *PaFT1* knock-down *Phalaenopsis* plants generated by virus-induced gene silencing exhibit delayed spiking. These studies suggest functional conservation of *FT* and *FD* genes, which may have evolved and integrated into distinct regulatory circuits in monopodial orchids, *Arabidopsis* and rice that promote flowering under their own inductive conditions.

## Introduction

Plants have evolved mechanisms to integrate environmental and developmental signals and precisely control the transition terminating vegetative growth and initiating the formation of flowers.

In *Arabidopsis*, flowering is triggered by multiple pathways [1] converging on a few integrators such as *FLOWERING LOCUS T* (*FT*) [2]. *FT* mRNA expression is induced in the leaves and its protein moves systemically to the shoot apical meristem where flowers bloom in response to long summer days (LDs) [3,4] indicating the FT protein acts as a major flowering hormone ‘florigen’ [5,6]. In addition, FT-like genes are well conserved among flowering plants and were reported to play a role of flowering activators in plants other than *Arabidopsis*, including tomato [7], squash [8] and rice [9,10]. The FT movement from the leaves to meristems requires interaction with a partner called FTIP, or FT interacting protein [11]. On reaching the meristem, FT interacts with a bZIP domain transcription factor, FD and together they activate expression of downstream genes that change the identity of the meristem to that of a flower. One example target of the FT-FD complex is *APETALA1* (*AP1*), a meristem identity gene; another target is *SUPPRESSOR OF OVEREXPRESSION OF CONSTANS1* (*SOC1*), which promotes the expression of another meristem identity gene called *LEAFY* (*LFY*). Both *AP1* and *LFY* expression are repressed during vegetative growth, and this repression is released upon arrival of FT in the meristem [12].

In rice, the expression of *Heading date3a* (*Hd3a*), a rice *FT* orthologue is up-regulated only under inductive short-day (SD) conditions [13] and 14-3-3 proteins act as intracellular receptors for Hd3a proteins. A hexameric florigen activation complex (FAC) composed of Hd3a, 14-3-3 proteins and OsFD1 activates *OsMADS15*, a rice homologue of *Arabidopsis AP1* leading to floral induction [10]. Another model species used to study SD flowering response, morning glory (*Pharbitis nil*), possesses two *FT* orthologues, *PnFT1* and *PnFT2* [14]. The expression of the genes exhibits circadian rhythms that are set by the onset of darkness and are upregulated at the end of the night under SD only if the night is sufficiently long. Despite the conserved functions of *FT* homologues, their expression appears to be controlled by different systems in different plant species.

*Phalaenopsis* is an epiphytic and monopodial orchid with thick and succulent leaves. It is an ornamental crop species of great economic value. In general, orchids can be divided into two groups, sympodial and monopodial, based on their growth morphology. Sympodial orchids such as those from the genera *Cattleya*, *Cymbidium*, *Dendrobium* and *Oncidium* grow from a stem that is horizontal. Monopodial orchids on the other hand, including *Phalaenopsis*, *Paphiopedilums* and *Vanda* grow vertically as a single upright stem with one leaf following another on opposite sides of the center. *Phalaenopsis aphrodite* subsp. *formosana*, a Taiwanese native *Phalaenopsis* orchid, is considered to be a model *Phalanopsis* species [15, 16]. Like many other flowering plants, the flowering of orchids is affected by several environmental factors such as photoperiod and temperature [17–19]. Most *Phalaenopsis* species are native to areas close to the equator and thus they do not need a specific photoperiod to induce flowering although specifics depend on the orchid genus and can even differ according to species. Instead, low ambient temperature is one of the triggers for the flowering initiation of *Phalaenopsis* orchids including *P*. *aphrodite* subsp. *formosana*. Also, orchids including *Phalaenopsisis* are crassulacean acid metabolism (CAM) plants like pineapples and cacti that can tolerate high temperatures using well-adapted metabolic strategies for growth [20]. Commercially, orchid seedlings are produced through embryo culture in vitro [21] or clonal propagation [22].

Spike induction (initiation of inflorescences) of *Phalaenopsis* was significantly inhibited when it was grown under a constant temperature higher than 28°C [23]. Conversely, diurnal fluctuation of high day and low night temperature or cool temperature in the night promoted spike induction [24–26]. Currently, many growers use very expensive air-conditioning to cool temperatures down to 18–25°C inside greenhouses to spike *Phalaenopsis* during the warm period of the year [27]. Therefore, it is important to functionally characterize flowering genes such as *FT* and *FD* in the orchid for better understanding of flowering under inductive conditions. Such understanding would also be beneficial for the production of new varieties which do not require cooling for flowering in the future. In *Arabidopsis*, it was reported recently that the abundance of the SHORT VEGETATIVE PHASE (SVP)-FLOWERING LOCUS M (FLM) complex, as a floral repressor of *FT* expression is regulated by ambient temperature [28, 29]. Interestingly, two MADS-box floral repressors, FLM-ß and SVP were reported to be down-regulated transcriptionally and post-transcriptionally by high ambient temperature [28].

The *Arabidopsis FLOWERING LOCUS C* (*FLC*) gene encodes a MADS domain protein that acts as a repressor of flowering by suppressing *FT* and *SOC1* [30, 31]. *FRIGIDA* (*FRI*) is a positive regulator of *FLC* [32]. Flowering of *Arabidopsis* is accelerated by prolonged exposure to cold (vernalization) and *FLC* levels progressively decline during the cold periods. Therefore, loss-of-function mutations in either *FRI* or *FLC* eliminate the vernalization requirement. Most commonly used lab strains of *Arabidopsis* such as Columbia lack active *FRI* and/or *FLC* alleles, and exhibit rapid-flowering behavior under inductive long days (LD) [32, 33]. In cereals, which lack *FLC*, the day length and vernalization are also likely to be interconnected through FT-like genes. In the cases of barley and wheat, the naturally occurring ‘vernalization genes’, *VRN-H3* and *VRN-B4* respectively, have been shown to encode *FT* orthologues [34]. In addition, *OnFT*, *PhFT* and *CgFT*, *FT* homologues from orchids such as *Oncidium* Gower Ramsey, *Phalaenopsis* hybrid Fortune Salzman and *Cymbidium goeringii*, respectively, were recently reported [35–37]. In *Arabidopsis*, distinct molecular mechanisms control *FT* expression for subsequent flowering under different ranges of environmental temperature. However, *Phalaenopsis* orchids including *P*. *aphrodite* subsp. *formosana* are known to originate from tropical and subtropical areas of the south pacific islands where photoperiod is almost constant throughout the year [23]. These orchids generally do not require vernalization despite low ambient temperature being necessary for flowering. Therefore, rather than photoperiod and/or vernalization, recognition and signaling systems for low environmental temperature are likely to be the major triggers for the induction of *FT* expression and consequently, the transition from vegetative to reproductive growth in *Phalaenopsis* orchids.

Here we demonstrated that *PaFT1* that encodes an orthologue of *Arabidopsis FT* was accumulated during the low ambient temperature treatment required for floral induction of the monopodial orchid *P*. *aphrodite* subsp. *formosana*. We further demonstrated that the functional role of *PaFT1* as a floral inducer is conserved in the orchid. Moreover, we showed that PaFT1-interacting protein, PaFD has a conserved floral activation function. Since plants have adapted themselves to various environments for successful reproduction, each plant has developed its own strategy to control the timing of floral transition. We provide evidence at least partially supporting the notion that distinct regulation of the FT and FD genes may have evolved for rapid flowering by inductive environmental cues in the monopodial orchid, *P*. *aphrodite* subsp. *formosana*.

## Materials and Methods

### Plant Materials and Growth Conditions

*Phalaenopsis* orchids (*P*. *aphrodite* subsp. *formosana*) at different developmental stages were purchased from Chain Port Orchid Nursery (Ping Tung, Taiwan). The orchids were adapted in the growth chamber environment (16 h light, 28°C/8 h dark, 25°C) for 2 weeks before starting each experiment. Wild-type *Arabidopsis* (*A*. *thaliana* ecotype Col) was used to generate transgenic plants. *ft-10*, *fd-3* [38], *soc1-2* [39] and *FRI*-Col [40] were described previously. *SOC1*:*GUS* and *35S*:*SVP* seeds were kind gifts from Dr. Ilha Lee (Seoul National University, Korea) and Dr. Peter Huijer (MPIZ, Germany), respectively. Generally, *Arabidopsis* plants were grown in the growth chamber under LD conditions (16/8-h photoperiod at 100 μmol m-2 s-1) at 23°C. Two *Japonica*-type rice cultivars, Dongjin, a Korean cultivar and Tainung67 (TNG67), a Taiwanese cultivar were used to produce transgenic rice plants and the transgenic plants were grown in the growth chamber or in the outdoor GMO greenhouse of the Academia Sinica Biotechnology Center in Southern Taiwan. For *Arabidopsis* flowering time measurement, 8 to 12 plants per line were counted for total leaf numbers when their first flowers were at anthesis. Days of heading of 8–12 rice plants per each line were measured when panicles were emerged.

### Cloning of *PaFT1* Gene from *P*. *aphrodite* subsp. *formosana*

Total RNA extracted from young spikes (≤ 2 cm in length) of *P*. *aphrodite* subsp. *formosana* was used for cDNA synthesis as described by Su et al [16] and Bilgin et al [41]. Synthesized cDNAs and degenerated primers were used for the amplification of *PaFT1*. Degenerated primers for *PaFT1* were as follows: forward primer (Deg F: 5′CHTTCTACACBCTYGTSATGGTAG3′) and reverse primer (Deg R: 5′CDGGSGCGTAMACYGTCTG3′). The amplified PCR product was cloned into pCR-Blunt II TOPO vector (Invitrogen) and the sequence of the partial *PaFT1* was verified to have high similarity with PEBP genes. Internal gene-specific primers for *PaFT1* were designed for the isolation of a full-length clone of *PaFT1* by RACE (Rapid Amplification of cDNA Ends) using SMART RACE cDNA amplification kit (BD Biosciences Clontech). Gene-specific primers for 5′ and 3′-RACE of *PaFT1* are 5′GAGGATCACTTGGACTTGGAGC3′ (GS-5′RACE) and 5′GTTGTTTCATCAACTAGGCCG3′ (GS-3′RACE), respectively. The cDNA of *PaFT1* full open reading frame (ORF) was obtained by PCR with *attB*-linked gene-specific primers and the *PaFT1* entry clone was constructed by BP reaction with pDONR201 (Invitrogen). The PaFT1Y86H form was generated by point mutagenesis using Stratagene’s QuikChange site-directed mutagenesis kit. The following two primers containing mismatched base pair (from T to C, bold type and underlined) were used for the mutagenesis: 5′CTCAACTTAGAGAACACTTACACTGGTTAG and 5'CTAACCAGTGTAAGTGTTCTCTAAGTTGAG.

### Promoter Isolation and GUS Expression

Genomic DNA of *P*. *aphrodite* subsp. *formosana* was isolated by DNeasy Plant Mini kit (Qiagen). Genomic clone of *PaFT1* was isolated by PCR with Phusion taq polymerase (NEB) and the exons and introns were annotated compared with the *PaFT1* cDNA sequence. The promoter region of *PaFT1* was isolated by inverse PCR (iPCR) together with the aid of Genome Walker Universal Kit (Clontech). The promoter entry clone was constructed and sequentially introduced into pMDC163 [42] (Curtis and Grossniklaus, 2003) to generate *PaFT1* promoter:GUS construction. For the expression of *PaFT1* genomic clone containing its own promoter and coding region, the pAlligator2 vector without *35S* promoter and the triple HA was used for the destination vector. GUS staining with *Arabidopsis* seedlings was performed as described by An et al [43].

### Expression Analyses

Total RNAs from various organs of *P*. *aphrodite* subsp. *formosana* were extracted by RNeasy Plant Mini Kit (Qiagen) and treated with RNase-free DNase (Invitrogen) following the manufacturer’s protocol to remove any residual genomic DNA. DNase-treated RNA was subjected to reverse transcriptase reactions using oligo-dT primer and Superscript II reverse transcriptase (Invitrogen) according to the manufacturer’s instructions. The same procedure was applied to cDNA syntheses of *Arabidopsis* and rice after RNA extraction. Subsequent PCR was performed with the first-strand cDNA mixture and EX-Taq polymerase (Takara Biochemical, Japan). Quantitative real time-PCR (qPCR) was performed on the CFX96TM real-time system (Bio-Rad) using Maxima SYBR Green qPCR Master Mix (Thermo). The primers used for quantification are listed in S1 Table. For PCR, each sample was analyzed in triplicate. The running protocol was: denaturation at 95°C for 10 min, annealing/extension repeated 40 times (95°C for 15 s and 60°C for 30 s, data acquisition was performed). Gene expression data were normalized to the expression of housekeeping genes. For the expressional analyses of *P*. *aphrodite* subsp. *formosana*, *PaACT* [16] and *PaUBQ* [16] genes were used for normalization but only the figures using *PaACT* are shown as both sets of results were similar. The primers used in this study are listed in S2 Table. At least two independent experiments were performed for RNA extraction in expressional analyses.

### *In Situ* Hybridization

Young spikes of *Phalaenopsis* orchid were collected and fixed, dehydrated, embedded, sliced (10 ìm thickness), and performed hybridization as previously described by Lin et al [44] with slight modification. For preparation of digoxigenin (DIG)-labeled RNA probes, we amplified gene-specific 261 bp fragment of *PaFD* using the following primers: 5'GTTCGTCCAACAGTCTTC3' (forward) and 5'GTTTCCAGACTTCTTCCATAC3' (reverse). The DNA fragment was cloned into pGEM-T vector (Promega) and each sense and antisense probe was synthesized by T7 and SP6 RNA polymerases, respectively. Hybridization was performed at 63°C or 66°C with 20 ng of DIG-labeled RNA probe.

### Yeast Two-Hybrid Screening for PaFD

*PaFT1* full-length ORF was cloned in-frame in the pBD-GAL4 Cam vector (Stratagene) to generate a pBD: *PaFT1* vector as a bait. For the cDNA library construction, total RNA was extracted from young spikes (≤ 2 cm in length) of *P*. *aphrodite* subsp. *formosana* and poly (A) + RNA was isolated using a PolyATract mRNA purification kit (Promega). The GAL4 AD vector library was constructed using a GAL4 Two-Hybrid Phagemid Vector Kit (Stratagene) according to manufacturer’s instructions. Screening and X-gal filter assay were performed as described previously [45].

The PaFDΔ1–53 clone was generated by PCR using the following *attB*-linked primers: 5'GGGGACAAGTTTGTACAAAAAAGCAGGCTGCATGGAAGAAGTCTGGAAACACATTGAC and 5’GGGGACCACTTTGTACAAGAAAGCTGGGTGTTAAAATGGCGCGGATGAAGTTCTCTGAAG. PaFD T225A, S226A, S227A and triple (PaFD T225A, S226A, S227A) clones were also generated by PCR using the following primers: 5' GGGGACAAGTTTGTACAAAAAAGCAGGCTGCATGTGGCTCCTATCTCCTGC (forward), 5' GGGGACCACTTTGTACAAGAAAGCTGGGTGTTAAAATGGCGCGGATGAAGCTCTCTGAAG (reverse for T225A), 5' GGGGACCACTTTGTACAAGAAAGCTGGGTGTTAAAATGGCGCGGATGCAGTTCTCTGAAG (reverse for S226A), 5' GGGGACCACTTTGTACAAGAAAGCTGGGTGTTAAAATGGCGCGGCTGAAGTTCTCTG (reverse for S227A), 5' GGGGACCACTTTGTACAAGAAAGCTGGGTGTTAAAATGGCGCGGCTGCAGCTCTCTGAAG (reverse for triple). Amplified PCR products were cloned into pDNONR201 *via* BP reaction (Invitrogen).

### Particle Bombardment and BiFC Assays

For cellular localization of AtFT, AtFD, AtFDP, PaFT1 and PaFD in *Arabidopsis*, either YFP:GW or CFP:GW vector was used for the florescence fusion as described previously [46]. For BiFC assays in *Arabidopsis*, *Arabidopsis FT* and *PaFT1* cDNA were cloned into pVYCE vector for AtFT:cYFP and PaFT1:cYFP fusions. *AtFD* and *AtFDP* cDNAs were cloned into pVYNE vector for nYFP:AtFD and nYFP:AtFDP fusions, respectively [47]. Bombardment on *Arabidopsis* leaves was carried out as described by Shirasu et al [48]. For BiFC in *Phalaenopsis*, cDNAs encoding the *PaFT1* and *PaFD* genes were introduced into pE3136 and pE3130, respectively [49] (http://www.bio.purdue.edu/people/faculty/gelvin/nsf/protocols_vectors.htm). Bombardment-mediated transient transformation of *Phalaenopsis* and generation of images were performed as described by Su et al [16].

### VIGS Assays

Each specific fragment of *PaFT1*, *PaFD* and *GUS* genes was cloned into pCymMV vector [50] by *in vitro* recombination with BP Clonase II (Invitrogen) to generate pCymMV-*GUS*, pCymMV-*PaFT1* and pCymMV-*PaFD*, respectively. For growth of *Agrobacterium* and leaf injection, we followed the procedure described by Hsieh et al [51, 52].

### Plant Transformation and Analyses of Transgenic Plants

For the p*SUC2*:*PaFT1* and p*KNAT1*:*PaFT1* constructs, the *PaFT1* entry clone was inserted into the p*SUC2*:Gateway (GW) and p*KNAT1*:GW destination vectors, respectively [43, 46]. For the p*FD*:*PaFT1* construct, the *35S* promoter and the triple HA of the pAlligator2 vector was exchanged for the 3.1-kb *FD* promoter and then *PaFT1* was introduced by LR reaction. With the same strategy, *PaFT1* was also fused to *Arabidopsis heat shock protein* (*HSP*) *18*.*2* promoter [53] to generate p*HSP18*.*2*:*PaFT1*. All plasmids for plant transformation were introduced into *Agrobacterium* strain GV3101 (pMP90RK) [54] and transformed into Columbia wild-type, *ft-10* or *ft-10 soc1-2* double homozygous plants by the floral-dip method [55]. For overexpression of target genes in rice, binary vector, pGA3426 was used and each transgene construct was introduced into rice genome by *Agrobacterium*-mediated transformation [56].

## Results

### Isolation and Molecular Characterization of *PaFT1*

We isolated *PaFT1* by a combined reverse transcription PCR (RT-PCR) and RACE strategy from young spikes of *P*. *aphrodite* subsp. *formosana*. Degenerated primers were designed based on the conserved regions of the *FT* sequences from *Oncidium* orchids, rice and barley (see the materials and methods).

The *PaFT1* cDNA encodes a 178 amino acid protein with a calculated molecular mass of 20.02 kDa and a theoretical pI of 6.83. The PaFT1 protein showed 70%, 76% and 89% identity to *Arabidopsis* FT, rice Hd3a and *Oncidium* orchid OnFT, respectively. The key amino acid residues Tyr and Gln that are conserved among the FT homologues were located at positions 86 and 141 of the PaFT1 protein (S1 Fig) [57–59].

The PaFT1 and other FT protein sequences from various plant species were used to construct a phylogenetic tree. The PaFT1 isolated from *P*. *aphrodite* subsp. *formosana* belonged to the FT family of monocotyledonous plants and interestingly, it was grouped with two other orchid FTs, *Oncidium* FT (OnFT) and *Cymbidium* FT (CgFT) (S1 Fig).

### Expression Pattern of *PaFT1*

**Fig 1:**
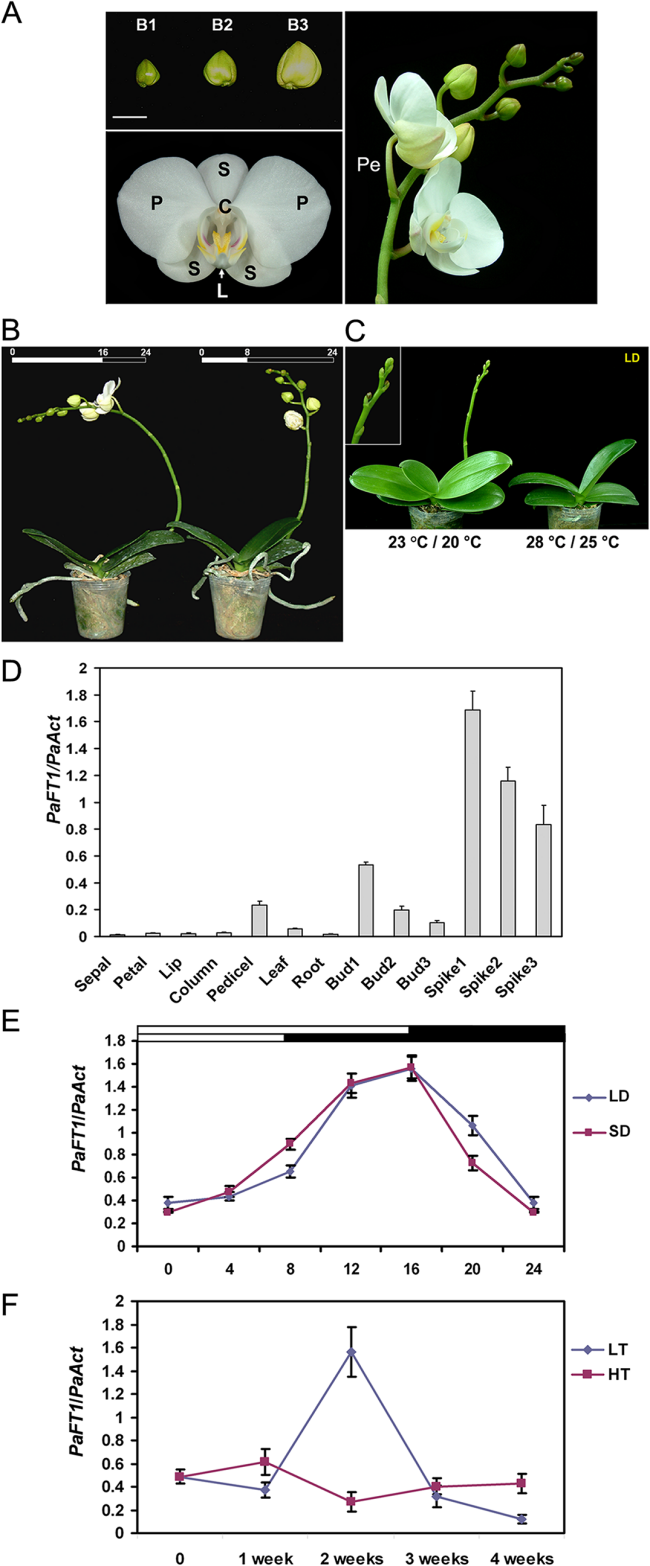
Growth and flowering of *P*.*aphrodite* subsp. *formosana* with the expression of *PaFT1*. A, Floral buds at different developmental stages and the structure of flower. S: sepal, P: petal, L: lip, C: column, Pe: pedicel. Bar is 1 cm. B, Spiking and flowering of *P*. *aphrodite* subsp. *formosana* under LD and SD conditions at constant 23°C. C, Under LD conditions, low temperature treatment is essential to induce inflorescence of *P*.*aphrodite* subsp. *formosana*. Day and night temperatures are shown in parenthesis. D, Spatial expression of *PaFT1* in *P*. *aphrodite* subsp. *formosana*. Materials for RNA extraction were harvested from six to eight plants. Bud 1, bud 2 and bud 3 indicate the B1, B2 and B3, respectively in A. Spike 1, spike 2 and spike 3 indicate ≤ 3 cm, 3–10 cm and ≥ 10 cm in length, respectively. The 3rd, 4th and 5th leaves were used for the leaf RNA extraction. E, Daily oscillation of *PaFT1* expression under LD and SD conditions. In each time point, leaves of 4 plants (18 months old) were harvested for RNA extraction. F, The effect of ambient temperature on *PaFT1* expression. LT; low temperature (23°C/20°C), HT; high temperature (28°C/25°C). Thirty six mature plants (34-month old as the stage 4) were grown at HT and then sixteen plants were transferred to the LT conditions. All leaves of four plants were used for the analysis of *PaFT1* expression, at each time point. All the samples were harvested at the end of light (ZT 16). Two independent experimental results showed similar expression patterns.

The spatial expression pattern of *PaFT1* was investigated by quantitative RT-PCR analyses. The *PaFT1* transcript accumulated to high levels in developing inflorescences (spikes) and developing floral buds, and was also detected in vegetative organs such as leaves and roots as well as reproductive organs such as lips, columns, pedicels. However, *PaFT1* mRNA was hardly detectable in sepals and petals (Fig 1).

To explore whether *PaFT1* expression oscillated over 24 hours, the *PaFT1* transcript was analyzed every 4 hours over a 24-hour period under both LD and SD conditions using leaves of 18-month-old orchids. The highest peak of *PaFT1* expression was detected at zeitgeber time (ZT) 16 irrespective of the photoperiod. The lowest level of expression of *PaFT1* was observed at dawn (Fig 1). Since cooling treatment is necessary for spiking of *P*. *aphrodite* subsp. *formosana*, the expression of *PaFT1* was investigated under two different conditions (Fig 1). There was no clear difference in spiking induction time between the plants grown under SD and LD (Fig 1).

When the mature plants (34 months old) grown at high temperature (28°C light/25°C dark, LD) were transferred to a cooler temperature (23°C light/20°C dark, LD), spike sprouting initiated 4 weeks after the transfer (Fig 1). However, the plants maintained at the high temperature did not develop spikes. During this period, we measured mRNA levels of *PaFT1* and three putative *SOC1* homologues from the orchid at weekly intervals. The experiment revealed that *PaFT1* expression was increased at the two-week point in the transferred plants but was not changed in the plants that remained at high temperature (Fig 1). The three putative *SOC1* homologues, *PaSOC1-1*, *PaSOC1-2* and *PaSOC1-3*, were all highly expressed at the spiking stage under low ambient temperature although they showed distinct expression patterns during the temperature shift (S2 Fig).

### *PaFT1* Promotes Flowering in *Arabidopsis* and Rice

**Fig 2:**
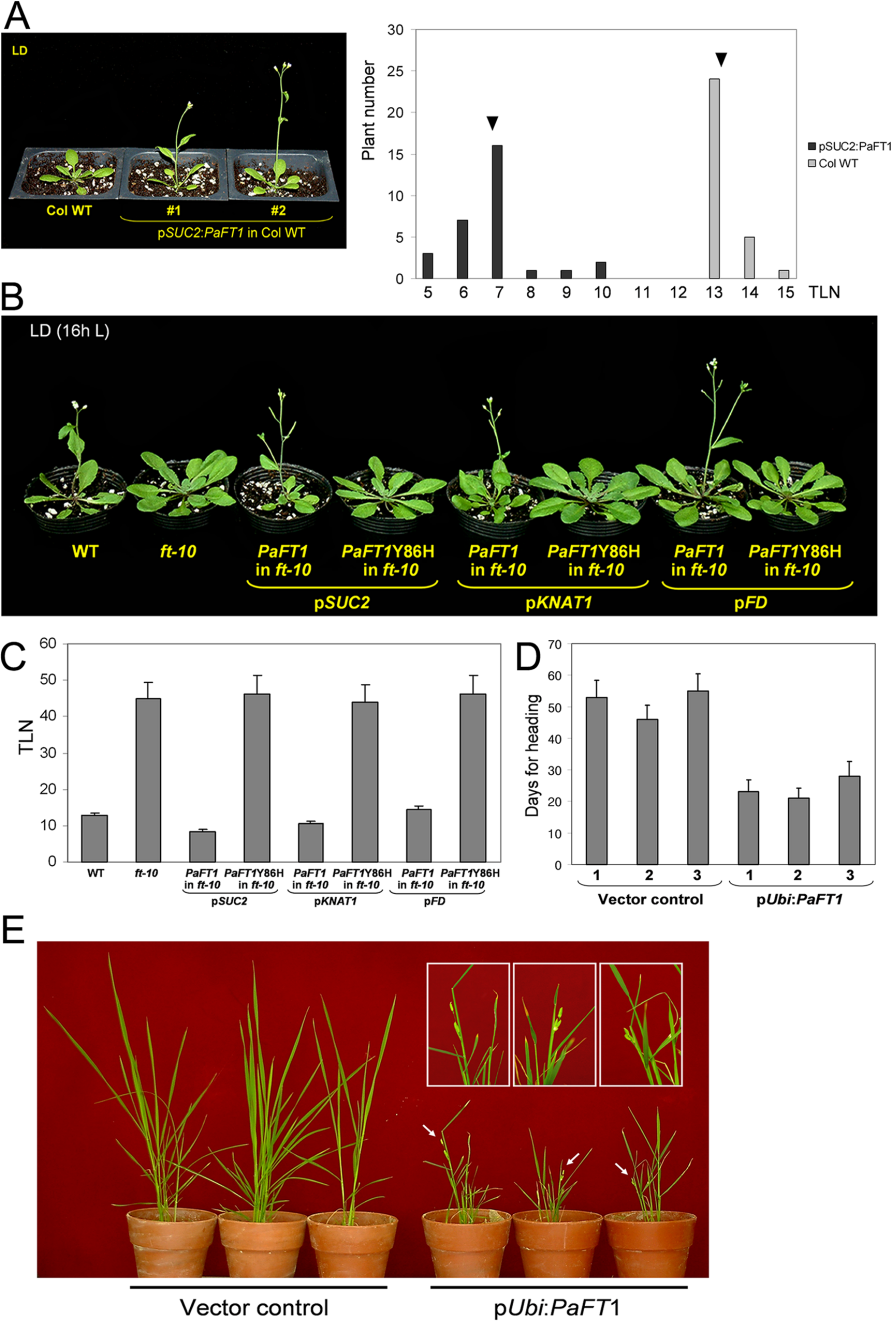
Phloem-specific expression of *PaFT1* in *Arabidopsis* and overexpression of *PaFT1* in rice drive early flowering. A, Comparison of flowering time between transgenic plants containing p*SUC2*:*PaFT1* and wild type plants. Thirty independent T1 plants were used for each genotype. Arrows represent the mean value of total leaf number in each genotype. P ≤ 0.0001 (Student’s *t*-test). B and C, *PaFT1* driven by phloem-specific or shoot apex-specific promoters rescues the late flowering phenotype of *Arabidopsis ft* null mutant, *ft-10*. This activity is at least dependent on Tyr-86 residue, one of the conserved amino acids among FT proteins from various plant species. D and E, Ectopic expression of *PaFT1* in rice also caused early flowering (Dongjin cultivar, grown under SD condition). Magnified panicles are shown in the box of E and flowering time data is shown in D. TLN means total leaf number.

To test the activity of *PaFT1* as a flowering regulator, the p*SUC2*:*PaFT1* transgene, which overexpresses *PaFT1* in the phloem companion cells, was introduced into *Arabidopsis* Columbia wild type (WT) plants. The transgenic plants exhibited early flowering with an average of 6.3 fewer leaves than the control WT (Fig 2).

**Fig 3:**
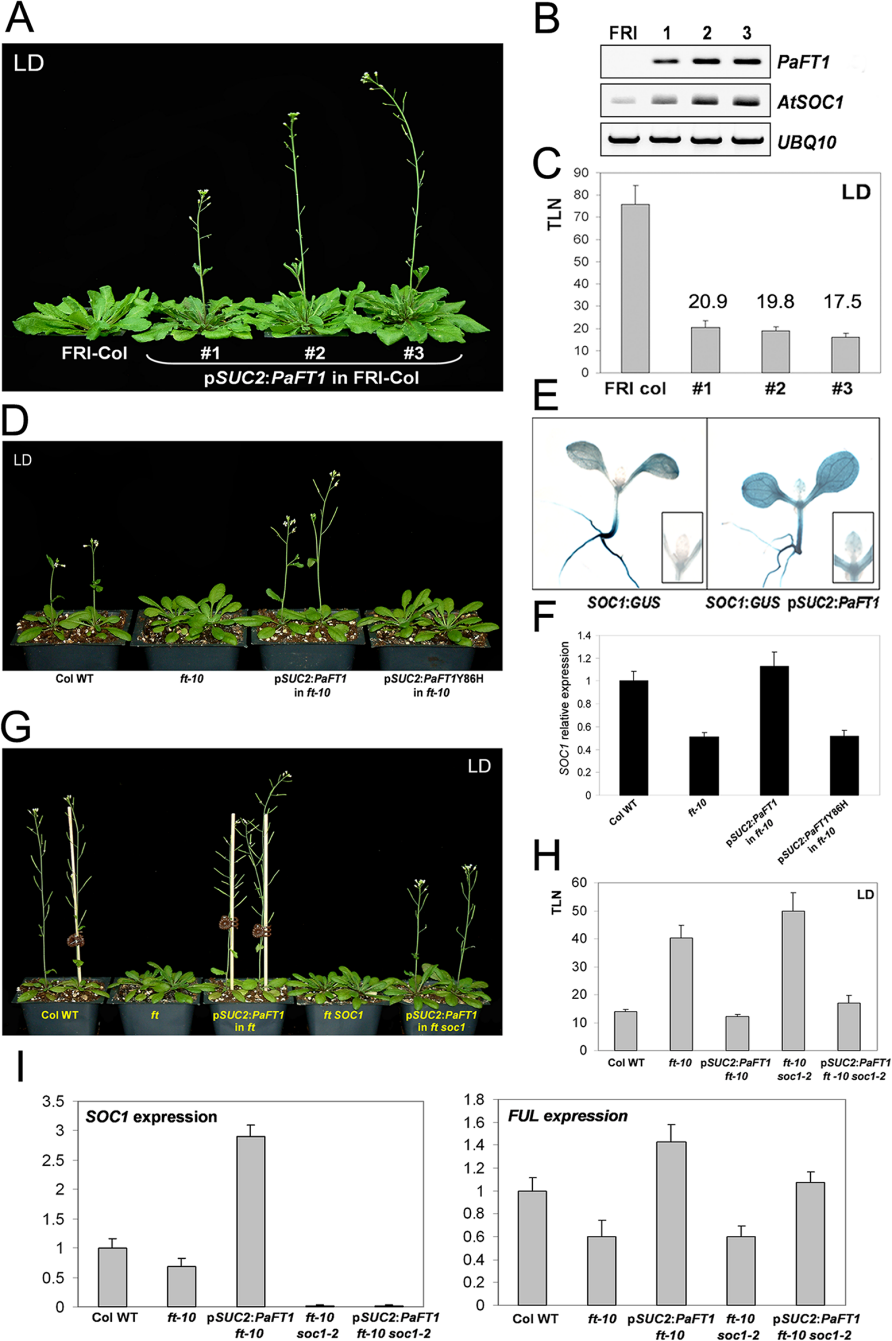
Effect of phloem-specific expression of *PaFT1* in *FRI* Col, *ft-10* and *ft-10 soc1-2* double mutant background. A and C, Phloem-specific expression of *PaFT1* overcomes the effect of *FRI*, a positive regulator of central floral repressor, *FLC*. C, Average total leaf numbers of three independent homozygous lines of p*SUC2*:*PaFT1* in *FRI*-Col were presented above each bar. B and E, *Arabidopsis SOC1* expression is increased in plants expressing *PaFT1* in phloem companion cells. D and F, The mutant form of PaFT1 loses the activity to increase the *SOC1* expression and induce flowering. G, H and I, The p*SUC2*:*PaFT1 ft-10 soc1-2* plants flower earlier than the double, *ft-10 soc1-2* implying that other factors are still affected by *PaFT1* in floral induction and *FUL* is one of the candidates. F and I, Relative expression was presented compared with that of Col WT.

In order to investigate whether *PaFT1* was able to rescue the late flowering phenotype caused by the *ft* mutation, the p*SUC2*:*PaFT1* construct was introduced into the *ft-10*, *ft* null mutant [60]. The transgenic *ft* mutant plants expressing *PaFT1* in the phloem companion cells flowered much earlier than the parental *ft* mutant plants with a similar total number of leaves to the WT (Fig 2). Expressing *PaFT1* under shoot apical meristem (SAM)-specific promoters such as p*KNAT1* or p*FD* also rescued the delayed flowering phenotype in the *ft* mutant (Fig 2). These results indicated that expression of *PaFT1* in phloem companion cells or SAM is sufficient to promote flowering in *Arabidopsis* plants that completely lack endogenous *FT*. However, a single amino acid change from the conserved Tyr-86 to His of PaFT1 resulted in a loss of the flowering promotion capability (Figs 2 and 3) [57–59].

We also introduced the *PaFT1* gene into Dongjin rice. *PaFT1* expression was under the control of maize *ubiqutin* promoter, a strong constitutive promoter in monocot plants [56]. Three independent transgenic lines containing p*Ubi*:*PaFT1* showed early heading compared with control plants containing the empty vector (Fig 2). Under SD condition, transgenic rice plants produced panicles within one month after sowing although they did not produce many healthy grains. This early flowering phenotype correlated with increased expression of two rice *AP1*-like genes, *OsMADS14* and *OsMADS15* (S3 Fig).

### Expression of *PaFT1* Overcomes the Late Flowering Phenotype of *FRI*-Col Plants

To test the contribution of *PaFT1* to the flowering of *FRI*-Col that contains an active *FRI* locus of San feliu-2 accession [61], the p*SUC2*:*PaFT1* construct was introduced into the *FRI*-Col.

Transgenic plants expressing *PaFT1* flowered earlier than control *FRI*-Col plants without cold treatment (Fig 3). As expected, *SOC1* expression was increased in the transgenic plants and the increase in *SOC1* transcripts was also observed in the *ft-10* mutants containing the p*SUC2*:*PaFT1* construct (Fig 3). However, PaFT1Y86H, a mutant form of PaFT1 could not induce *SOC1* expression (Fig 3).

To confirm whether *PaFT1* positively regulates *SOC1*, we employed a β-glucuronidase (GUS) reporter assay. We used p*SOC1*:*GUS* [60] plants to visualize the expression of *SOC1*. p*SUC2*:*PaFT1* p*SOC1*:*GUS* plants were produced to examine whether *SOC1* promoter-driven expression of *GUS* was affected by p*SUC2*:*PaFT1*. A histochemical *GUS* assay showed stronger GUS staining in the shoot apex, cotyledons and the first true leaf in the p*SUC2*:*PaFT1* p*SOC1*:*GUS* plants compared with p*SOC1*:*GUS* plants (Fig 3). This suggests that *SOC1* expression was reinforced by *PaFT1* and the increased *SOC1* expression contributed to the early flowering.

The p*SUC2*:*PaFT1* transgene was introduced into *ft-10 soc1-2* double mutants to evaluate the effect of the transgene in flowering of the double mutants. The p*SUC2*:*PaFT1 ft-10 soc1-2* plants flowered earlier with approximately 32 fewer leaves than the *ft-10 soc1-2* double mutants, whereas they flowered later with approximately 5 more leaves than the p*SUC2*:*PaFT1 ft-10* plants under LDs (Fig 3). This implies that *SOC1* is not a unique target of *PaFT1* for promotion of flowering in *Arabidopsis*. Recently, *soc1 ful* was reported to be epistatic to p*SUC2*:*FT* in flowering [62]. Analyses of *FRUITFULL* (*FUL*) transcript levels revealed that the gene expression was higher in p*SUC2*:*PaFT1 ft-10 soc1-2* plants compared to that in *ft-10 soc1-2* but lower than that of p*SUC2*:*PaFT1 ft-10* plants (Fig 3).This indicates that *PaFT1* induces *FUL* expression and sequentially promotes flowering in *Arabidopsis* lacking endogenous *FT* and/or *SOC1*.

### Expression of *PaFT1* Reduces the Effect of p*35S*:*SVP* in Flowering

**Fig 4:**
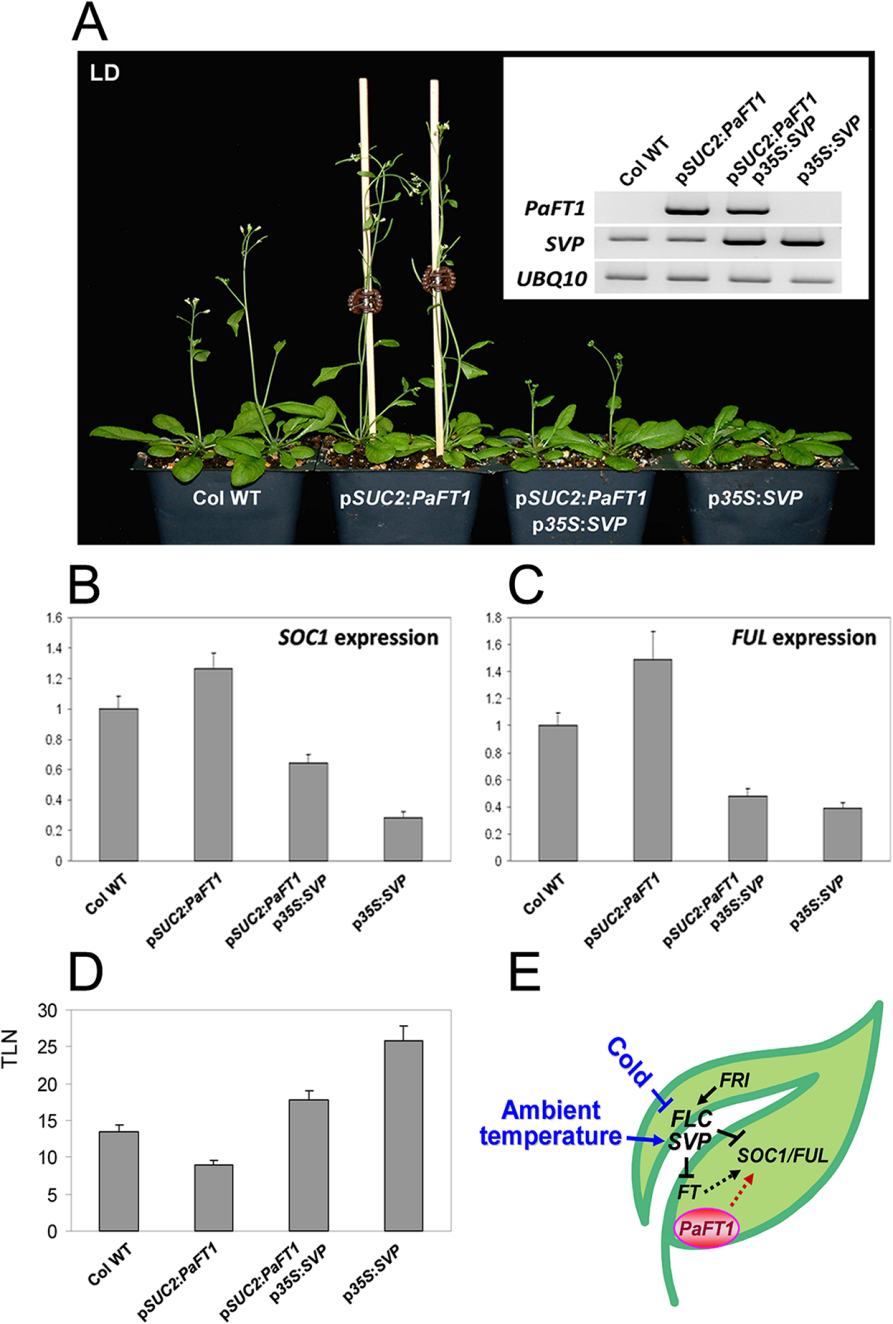
Phloem-specific expression of *PaFT1* reduces the late flowering effect by overexpression of *SVP* encoding an ambient temperature-dependent floral repressor. A and D, p*SUC2*:*PaFT1* retards the effect of late flowering by p*35S*:*SVP*. B and C, The expression of *SOC1* and *FUL* in each genotype shown in A. Relative expression was presented compared with that of Col WT. E, A model showing the effect of *PaFT1* expression in *Arabidopsis* flowering.

Since *SVP* has been identified as a flowering repressor mediated by ambient temperature, we generated p*SUC2*:*PaFT1 *p*35S*:*SVP *plants by introducing the transgene p*SUC2*:*PaFT1* into the p*35S*:*SVP* background to investigate whether phloem-specific expression of *PaFT1* is able to overcome the flowering repression effect of p*35S*:*SVP*. We observed that the expression level of *SOC1* and *FUL* was higher in the plants expressing *PaFT1* than in the plants expressing *SVP* alone (Fig 4). The p*SUC2*:*PaFT1* p*35S*:*SVP* plants flowered with an average of 8 fewer leaves than p*35S*:*SVP* plants under LD condition demonstrating that the phloem-specific expression of *PaFT1* partially suppresses SVP overexpression in flowering induction (Fig 4) although aberrant floral morphology was observed both in p*35S*:*SVP* [63] and p*SUC2*:*PaFT1* p*35S*:*SVP* plants (data not shown).

### Genomic Clone of *PaFT1* Partially Rescues the Late Flowering Phenotype of *ft-10*

**Fig 5:**
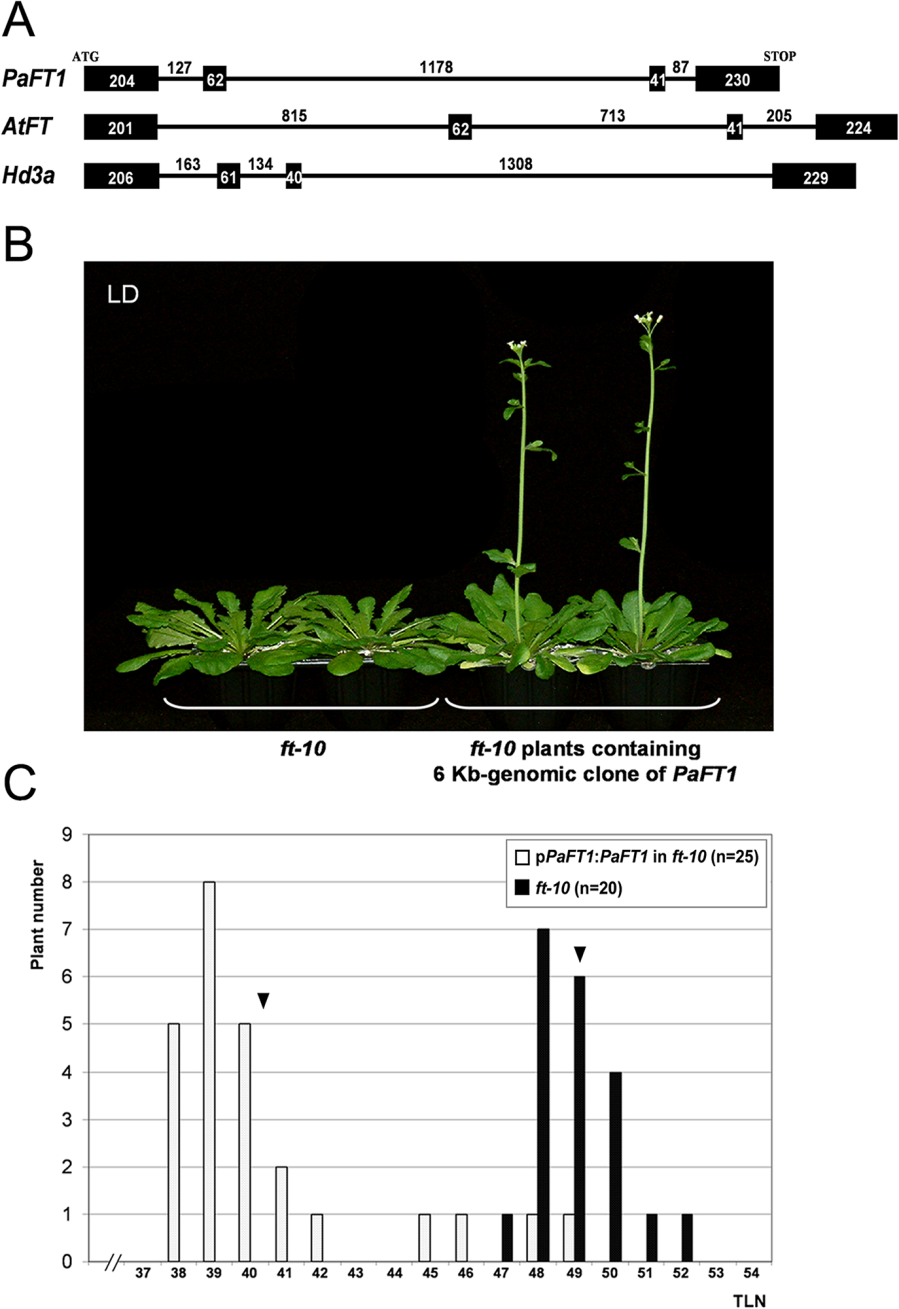
Genomic *PaFT1* partially complements *Arabidopsis ft* mutants. A, Genomic structure of *PaFT1*. *AtFT* is an *Arabidopsis FT* and *Hd3a* is a rice *FT* homologue. Filled boxes indicate coding sequences and the lines between boxes represent introns. Numbers represent the length of nucleotides in coding regions and introns (bp). B and C, 6-kb genomic clone of *PaFT1* containing its 4-kb promoter region partially rescues the late flowering phenotype of *Arabidopsis ft* null mutants. C, Flowering time was measured with twenty five individual T1 plants containing the genomic clone in the *ft* mutant background and twenty *ft-10* plants. Arrows represent the mean value of total leaf number in each genotype. P ≤ 0.0005 (Student’s *t*-test).

To evaluate whether the *PaFT1* genomic clone is functional in *Arabidopsis*, we isolated an approximate 6-kb fragment of *PaFT1* containing a 2-kb coding region and a 4-kb promoter region. The *PaFT1* gene consists of four exons and three introns in the ORF region similar to the *Arabidopsis FT* and rice *Hd3a* genes (Fig 5). The isolated genomic clone was introduced into *ft-10* and the flowering time of 25 individual T1 plants was measured (Fig 5). The transgenic plants produced flowers with an average of 9 fewer leaves than *ft-10* mutants under LD conditions. This indicates that the 4 kb-*PaFT1* promoter is, at least partially, functional and the orchid introns are properly spliced in *Arabidopsis* to produce functional PaFT1 proteins.

### Expression Pattern of p*PaFT1*:*GUS* in *Arabidopsis*

**Fig 6:**
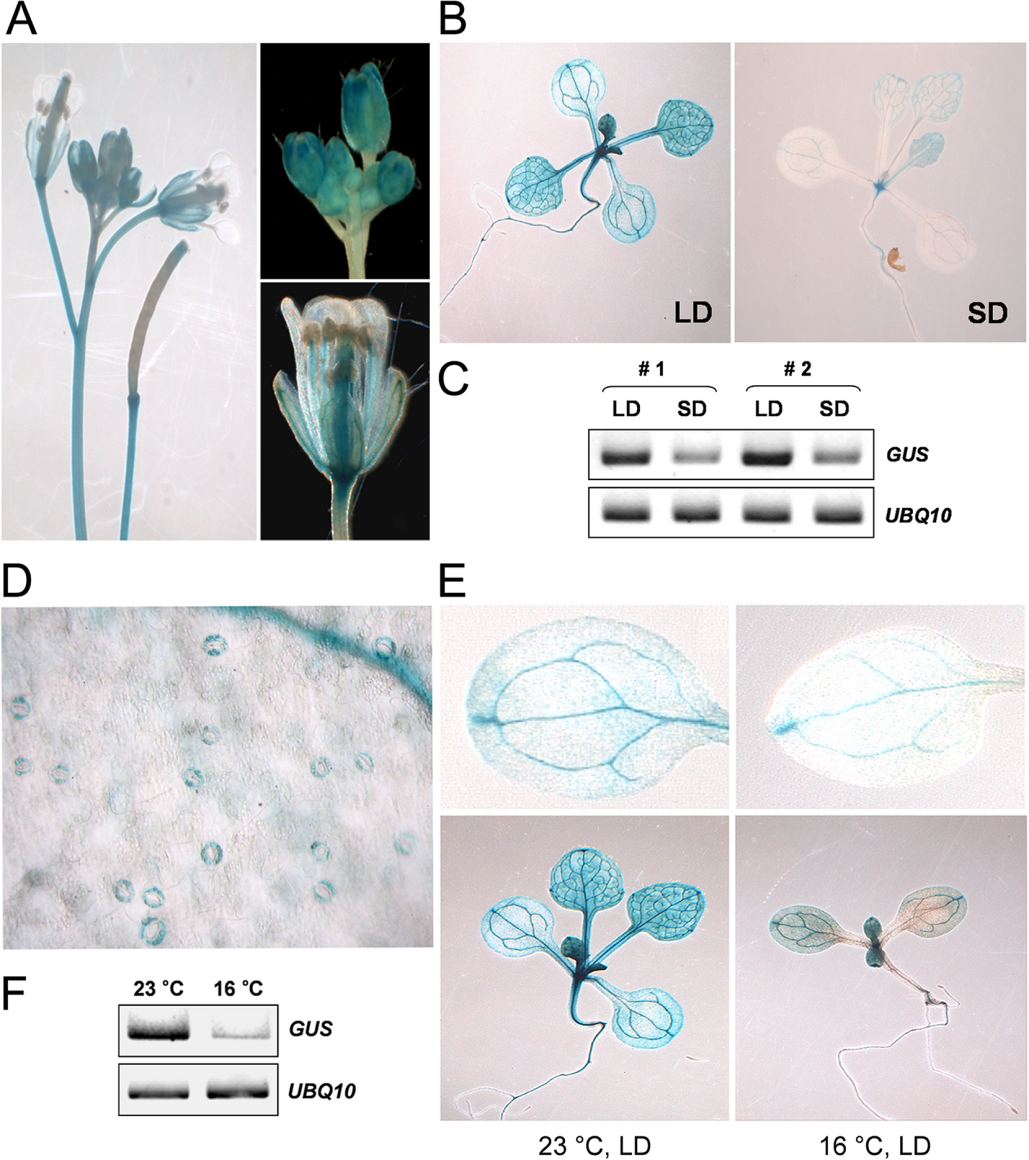
A homozygous line expressing a *PaFT1*-promoter GUS fusion that showed a characteristic staining pattern was chosen for histochemical analysis of p*PaFT1*:GUS expression in different organs of *Arabidopsis*. A, Inflorescence, floral buds, flower and siliques. B, 8-day-old seedlings grown under LD and 14-day-old seedlings grown under SD. C, *GUS* mRNA expression under different photoperiods. D, GUS expression in guard cells. E and F, Different levels of GUS expression are detectable under different temperature. 8-day-old seedlings grown under constant 23°C and 16°C, respectively were used for GUS staining or mRNA analysis.

Because the *PaFT1* promoter is functional in *Arabidopsis*, we created a *PaFT1* promoter reporter line, p*PaFT1*:*GUS* using the 4-kb promoter of *PaFT1*. The GUS expression was detected in vasculature and guard cells in the same way as observed in *Arabidopsis* FT (Fig 6) [64, 65]. However, strong GUS signals were also detectable in the shoot apex, which is distinguishable from the *Arabidopsis* FT expression pattern (Fig 6) [64]. The GUS signals were not present in the petals and mature anthers (Fig 6). The GUS expression was regulated by photoperiod and growth temperature. Under LDs, *GUS* transcripts were highly accumulated and the GUS signal was also stronger than those from plants grown under SDs (Fig 6). Plants grown at 16°C showed reduced GUS expression compared with plants grown at 23°C under LDs (Fig 6). Thus, the response of *GUS* expression to different photoperiod and growth temperature displayed similar patterns to those of the *Arabidopsis FT* gene implying the existence of transacting factors acting on the both *FT* promoters from *Arabidopsis* and orchid.

### Induced Expression of *PaFT1* by High Temperature Causes Early Flowering in *Arabidopsis*

Since *Phalaenopsis* orchids did not respond to chemicals such as gibberellic acids in spiking when it was sprayed [66], other methods rather than chemicals were explored for the use in the inducible gene expression system in the orchid.

To establish heat-inducible flowering in orchid, we created p*HSP*18.2:*PaFT1* construction and introduced it into the *Arabidopsis ft-10* mutants. The transgenic plants did not display an early flowering phenotype under normal temperatures. However, heat treatment induced early flowering of the transgenic plants (S4 Fig) which resembled *Arabidopsis FT* [67]. A similar phenotype was observed in wild-type *Arabidopsis* containing p*HSP*18.2:*PaFT1* under SD condition with heat treatment (S4 Fig). This indicates that *PaFT1* is a floral activator and the *HSP*18.2 promoter has the potential to be used for heat-induced flowering in the orchid.

### PaFD Is a bZIP Domain Protein that Interacts with PaFT1 in Orchids

**Fig 7:**
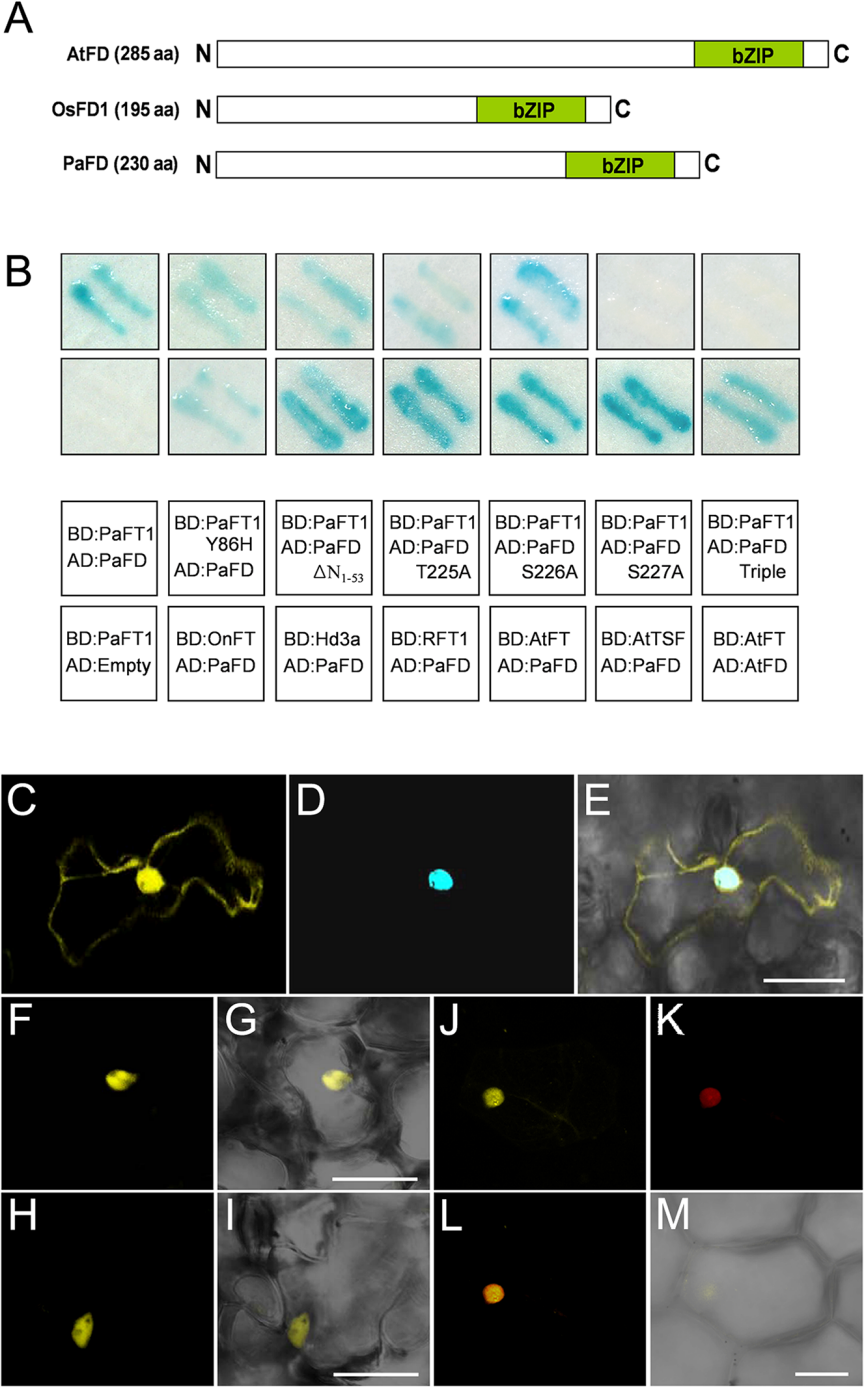
PaFD, a PaFT1-interacting bZIP protein. A, Comparison of PaFD with other FD proteins from *Arabidopsis* and rice. Green boxes indicate bZIP domain and the number in the parenthesis is the length of each polypeptide. B, PaFT1 interacts with PaFD in the yeast system. PaFD also interacts with FT proteins from other species such as *Arabidopsis* (AtFT, AtTSF), rice (Hd3a, RFT1) and *Oncidium* orchid (OnFT). PaFT1Y86H indicates mutant form of PaFT1 and PaFDΔN1-53, PaFD T225A, S226A, S227A indicates N-terminal deletion and mutant forms of PaFD, respectively. The interaction between AtFT and AtFD is a positive control. C, YFP:PaFT1 fusion proteins in *Arabidopsis* cell. D, CFP:PaFD fusion proteins in *Arabidopsis* cell. E, Merged image of YFP:PaFT1 and CFP:PaFD proteins in *Arabidopsis* cell. F, G, H and I, BiFC assays in *Arabidopsis* cells. Plasmids for YFPn:PaFT1 and YFPc:PaFD expression were introduced into *Arabidopsis* cells, simultaneously. J, L and M, BiFC assays in *Phalaenopsis* cells. K, NLS:RFP was used for nuclear localization marker. Bar is 40 ìm in E, G, I and 20 ìm in M.

To investigate whether PaFT1 interacts with bZIP domain proteins in orchids in the same way as *Arabidopsis* FT or rice Hd3a, we performed a yeast two-hybrid screen using a young spike cDNA library to isolate a *P*. *aphrodite* homologue of *Arabidopsis* FD, as an interacting protein of PaFT1. Three partial and two full-length *PaFD* ORF clones were isolated. For verification of the interaction, we generated an entry clone for *PaFD* and sequentially produced a pAD:*PaFD*, a prey construction *via* LR recombination [38]. We observed that PaFT1 interacted with PaFD that consists of 230 amino acids containing the conserved bZIP domain at its carboxyl terminal similar to AtFD and OsFD1 (Fig 7). Interestingly, PaFD was also able to interact with FT proteins from other plant species such as *Arabidopsis*, rice and *Oncidium* orchid (Fig 7). Additionally, removal of the serine-rich amino terminal of PaFD (PaFDΔN1-53) had no effect on the interaction with PaFT1, while change of a single amino acid (S227A) or triple amino acids (T225A, S226A, S227A) prevented the interaction (Fig 7). In AtFD protein, the threonine residue at the 282nd position is important for AtFT-AtFD interaction: changing the 282nd threonine residue to alanine prevents its interaction with AtFT but changing it to serine does not affect the interaction [68]. When the 227th serine residue of PaFD, a positional equivalent of the 282nd threonine of AtFD was mutated to alanine, the PaFT1-PaFD interaction was also abolished (S9 Fig) indicating phosphorylation at the residue, threonine or serine may be critical for the interaction.

PaFT1 was localized both in the nucleus and cytoplasm which is similar to the subcellular localization of AtFT in plant cells (Fig 7). PaFD was exclusively localized in the nucleus as a putative bZIP transcription factor (Fig 7). Positive interaction was observed between PaFT1 and PaFD in *Phalaenopsis* cells as well as *Arabidopsis* cells *via* BiFC assays (Fig 7). A higher interaction affinity of AtFT to AtFD was observed than that of PaFT1 to AtFD and/or AtFDP based on florescence intensities in BiFC assays (S5 Fig).

### Expression of *PaFD* in the Shoot Apex Partially Complements *Arabidopsis fd* Mutants

**Fig 8:**
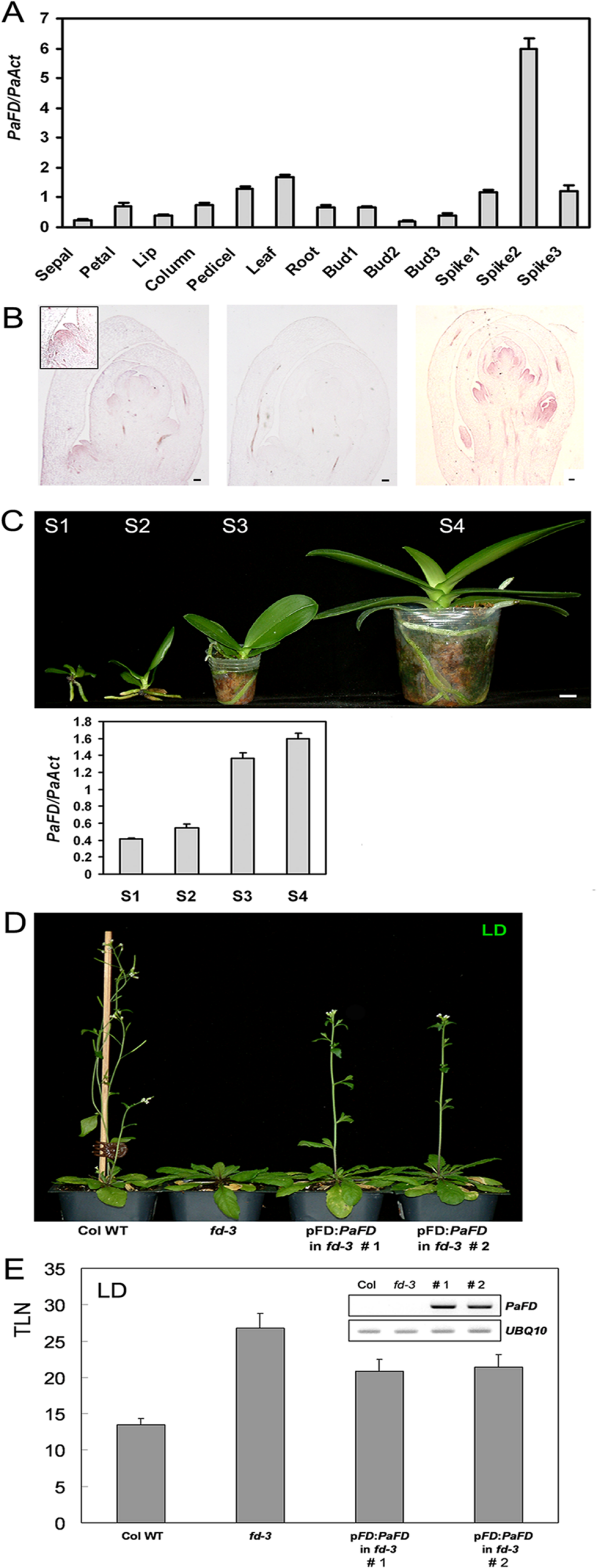
Spatiotemporal expression pattern of *PaFD* and its functional activity in *Arabidopsis*. A, Developmental stages of floral buds and spikes are described in Fig 1. B, *PaFD* expression in a developing spike by *in situ* hybridization. The magnified axillary floral meristem in the emerging spike (≤ 3 cm in length) is in the box (left panel) and the middle panel is a negative control with a sense probe. *PaFD* transcript was detected in the floral meristems in the developing spike (3–10 cm in length, right panel). Bar is 100 ìm. C, Expression pattern of *PaFD* in different developmental stages. The stage 1 (S1) is 16-month and the S2 is 20-month old stages of the orchid in the flasks. S3 and S4 show 26-month and 34-month old stages of the orchid in the pots, respectively. Bar is 1 cm. D, Flowering phenotypes of wild type, *fd* mutant (*fd-3*), and two independent homozygous transgenic plants expressing *PaFD* under the control of *Arabidopsis FD* promoter in *fd-3* background. E, Flowering time of the each genotype shown in D. Twelve individuals for each genotype were used for flowering time measurement and *PaFD* expression is detectable only in the transgenic plants.

*PaFD* transcripts were detectable in almost all organs in the orchid and they were gradually accumulated as plants become mature under LDs at high growth temperature (28/25°C, light/dark) (Fig 8 and S7 Fig). In particular, *in situ* hybridization results showed *PaFD* transcripts were accumulated in the emerging floral meristems in young spikes (Fig 8).

To test whether *PaFD* is a functional homologue of *Arabidopsis FD*, we constructed p*FD*:*PaFD* for the expression of *PaFD* under the control of *Arabidopsis FD* promoter and the p*FD*:*PaFD* transgene was introduced into *Arabidopsis fd-3* mutants *via* an *Agrobacterum*-mediated dipping procedure. Transgenic *fd-3* plants expressing *PaFD* driven by *Arabidopsis FD* promoter flowered with 5–6 fewer leaves than *Arabidopsis fd-3* under LD conditions demonstrating that *PaFD* plays a positive role, at least weakly, in *Arabidopsis* flowering in a similar manner to *Arabidopsis FD* (Fig 8). A higher level of *Arabidopsis AP1* and *SOC1* expression was also detected in the transgenic plants than in *fd-3* (S8 Fig).

### Ectopic Expression of *PaFD* Causes Early Heading in Rice

**Fig 9:**
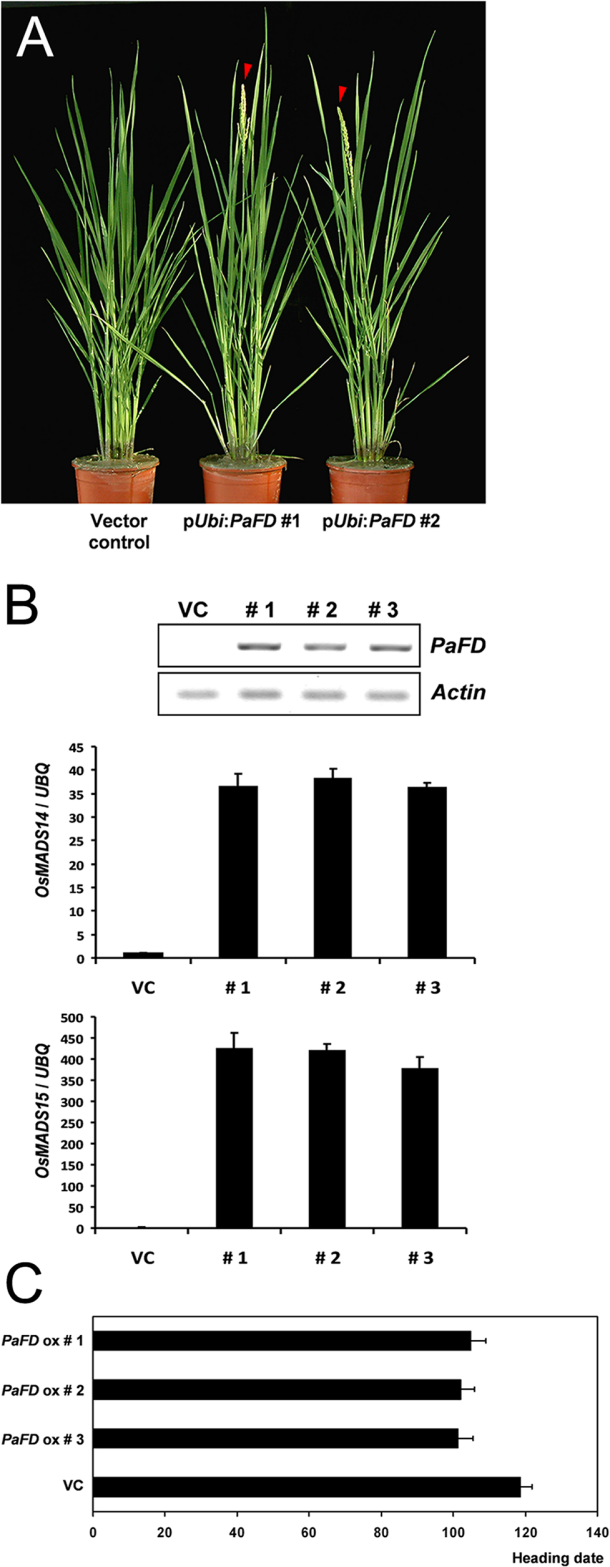
Ectopic expression of *PaFD* causes early heading in rice. A and B, The vector control plant is a transgenic rice plant containing an empty vector. Expression level of two rice *AP1* homologues, *OsMADS14* and *OsMADS15* is high compared with the control. VC indicates vector control. C, Flowering time (heading date) of the transgenic rice plants overexpressing *PaFD* compared with the control containing empty vector. Eight to twelve individual plants per each line were used for heading date measurement.

To examine the effect of *PaFD* in a monocotyledonous, SD plant, transgenic rice plants overexpressing *PaFD* were generated. Compared with control plants containing an empty vector, *PaFD* overexpressors showed early heading and the expression of rice *AP1* homologues, *OsMADS14* and *OsMADS15* was also increased in the transgenic rice plants (Fig 9). This observation implies that our orchid *FD* is able to induce flowering in rice through the increased expression of rice *AP1* genes. Overexpression of the rice *AP1* genes such as *OsMADS14* and *OsMADS15* has been reported to cause extremely early flowering in rice [69, 70]. It is noteworthy that two rice *FD* genes, *OsFD1* and *OsFD2* which belong to the *poaceae* FD group (S9 Fig), did not show alterations in heading time when their expression was increased or decreased indicating functional divergence between FDs that belong to different groups [10, 71].

### Reduced Expression of *PaFT1* in *P*. *aphrodite* subsp. *formosana* Causes Delayed Spiking

**Fig 10:**
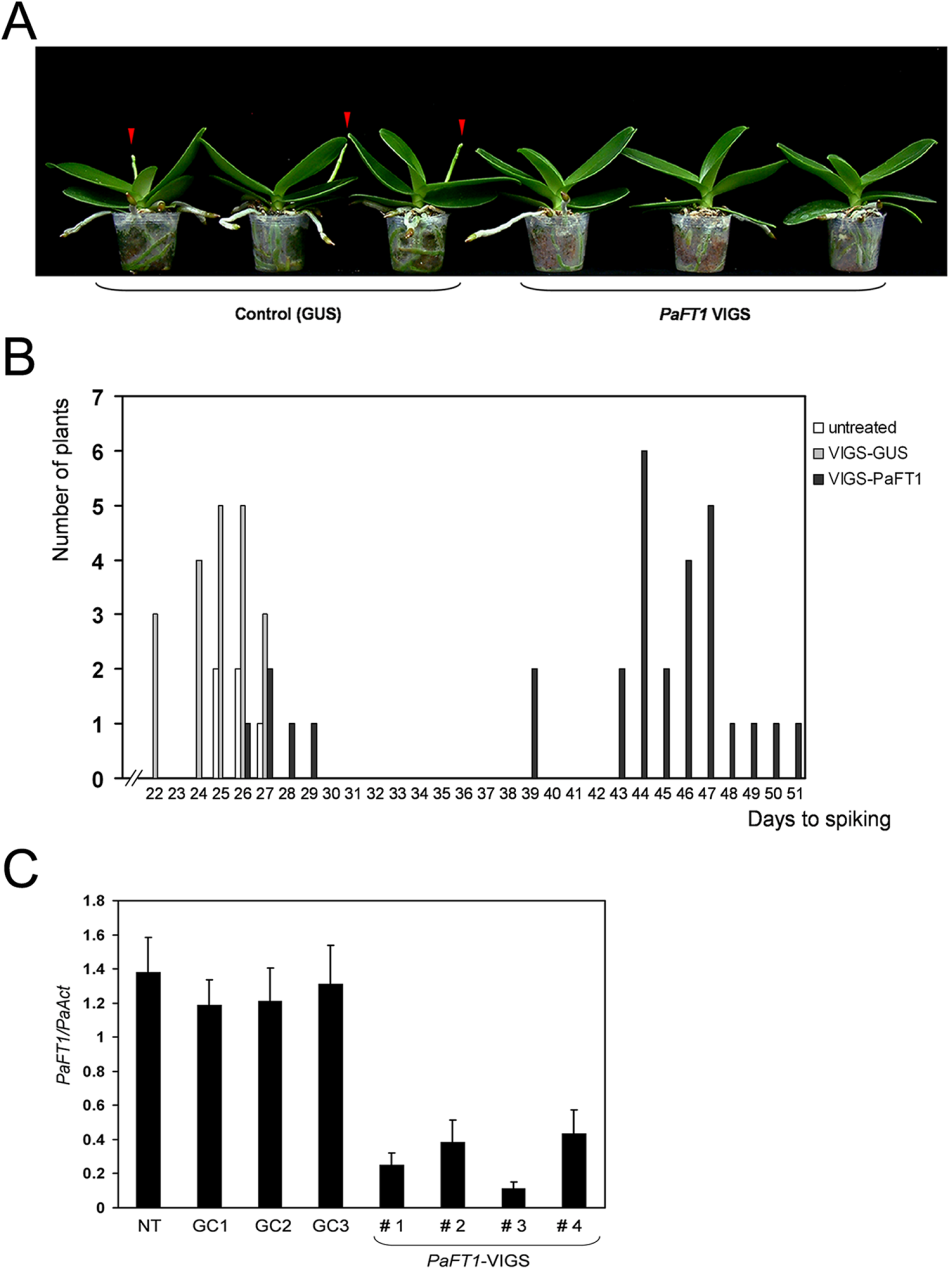
The VIGS of *PaFT1* exhibits delayed spiking. A and B, Compared with control lines for the VIGS of *GUS* gene, the VIGS of *PaFT1* lines show late flowering. B, Spiking time was measured in thirty independent lines for VIGS of *PaFT1* together with twenty lines for VIGS of *GUS* and five non-treated lines. We counted the first day of transferring the orchids to the low temperature condition (23°C / 20°C) after VIGS treatment as day one for spiking. C, Reduced expression of endogenous *PaFT1* in the VIGS lines of *PaFT1*. NT and GC mean non-treated and *GUS* control (VIGS of *GUS* gene), respectively.

Because a transformation system for *P*. *aphrodite* subsp. *formosana* has not been established, we applied the virus induced gene silencing (VIGS) method to study the *PaFT1* gene function in the orchid. We used the *GUS* gene as a control for VIGS and twenty individual orchids treated with VIGS of *GUS* flowered synchronously together with untreated plants under LDs with constant 23°C. However, orchids treated with VIGS of *PaFT1* exhibited significantly delayed spiking under the same growth conditions (Fig 10). The level of endogenous *PaFT1* expression in the VIGS of *PaFT1* orchids was reduced compared to that of controls (Fig 10). Thus, the reduced *PaFT1* expression contributes to the late spiking phenotype of the *PaFT1* VIGS orchids at spike-inducing temperature. Sequentially, the elongation of inflorescences, floral bud formation and opening of the flowers in the *PaFT1* VIGS orchids were all delayed compared with non-treated and *GUS* VIGS orchids (S10 Fig).

## Discussion

Functional study of the *FT* and *FD* genes of *P*.*aphrodite* subsp. *formosana* was conducted to understand floral induction of the orchid under its inductive environmental conditions. The diurnal expression pattern of *PaFT1* is similar under both LD and SD conditions (peaks at ZT 16) correlating with the similar flowering time of the orchid under the two different photoperiodic conditions. These results demonstrate that *P*. *aphrodite* subsp. *formosana* has a day-neutral flowering characteristics under our conditions. In the day-neutral plant tomato, expression of the *FT* orthologue *SINGLE FLOWER TRUSS* (*SFT*) is not regulated by the photoperiod, although overexpression of *SFT* promotes early flowering and *sft* mutations delay flowering [7]. Amino acid comparison and the construction of a phylogenetic tree suggest that PaFT1 is potentially an orthologue of FTs from various plant species that may control floral initiation in *P*. *aphrodite* subsp. *formosana*. The induction of expression of *PaFT1* during low ambient temperature treatment also supports this notion since flowering of the orchid was observed only under low ambient temperature. The regulation of *FT* expression by ambient temperature has been reported in other plant species such as *Arabidopsis* and rice. For example, *Arabidopsis* flowers earlier when grown at 23°C than at 16°C and rice flowers earlier at 27°C than at 23°C. Both plant species flower earlier and their *FT* expression is also higher when they are grown at high ambient temperature [72, 73] indicating a direct relationship between the level of *FT* expression and flowering time at a certain temperature. However, *PaFT1* expression is induced under low ambient temperature that mimics a mild winter season in the original habitat of the orchid. Recently, Hsu *et al*. demonstrated that *FT1* of poplar induces flowering in response to vernalization rather than photoperiod [74]. Indeed, in a naturally growing poplar tree, *FT1* transcript is abundant only in winter. Another example showing the relation between reduced temperature and accumulated *FT* transcripts was demonstrated in citrus stem as a result of floral induction by low temperature [75]. On the contrary, *NtFT* a homologue of the *FT* gene from *Narcissus tazetta* was reported to be expressed under high temperature and the floral transition of the plant was shown to be affected by high temperature but not by photoperiod or vernalization [76]. Thus, change in environmental temperature is sufficient to trigger floral initiation. However, whether known floral repressors such as SVP and FLM-ß regulate *PaFT1* expression in the orchid remains to be seen. The highest expression level of *PaFT1* in the young spikes may indicate that *PaFT1* accumulated in the sprouting inflorescence and the expression pattern of *PaFT1* in floral buds was similar to that of *OnFT*, a *FT* homologue from *Oncidium* Gower Ramsey [35] but different from that of *Arabidopsis FT* which, in contrast, gradually increased during flower maturation. Interestingly, *PaFT1* mRNA was relatively abundant in the pedicel, the connecting organ between the inflorescence and flower suggesting that *PaFT1* may play a pivotal role either in the initiation of the inflorescence (spiking) or in the formation of floral buds along the inflorescence or have a role in both steps. However, the expression level of *PaFT1* in roots was relatively low, similar to *OnFT* from *Oncidium* and *CgFT* from *Cymbidium*, but different from *PhFT*, a *FT* homologue of *Phalaenopsis* hybrid Fortune Salzman [35, 36, 37]. These results show that *FT* genes have distinct spatial expression among different orchid species. AtFT is closely related to the floral repressor, TERMINAL FLOWER1 (TFL1). Tyrosine (Tyr) at position 86 of PaFT1 is conserved in FT proteins from other plant species and important for the structural feature of FT as a floral activator [57–59]. Since substitution of Tyr at position 86 to His abolished its function as a floral activator and phloem-specific as well as shoot apex-specific expression of *PaFT1* in the *ft* mutant background complemented the mutant phenotype in flowering, PaFT1 is also likely to move from companion cells to the shoot apex where *AtFD* is expressed to induce flowering in *Arabidopsis*. Mild flowering phenotype of *Arabidopsis* plants expressing *PaFT1* is likely to be due to weaker interaction affinity between PaFT1 and AtFD than that of the AtFT-AtFD complex resulting in partial activation of down-stream genes or lack of recruitment of co-factors required for the activation of down stream targets for floral induction [59]. Early heading observed in transgenic rice plants overexpressing *PaFT1* demonstrates that *PaFT1* acts as a floral activator both in dicot LD plant, *Arabidopsis* and monocot SD plant, rice. The effect of *PaFT1* overexpression on rice heading was weaker than that of rice *Hd3a* overexpression since overexpression of *Hd3a* caused extremely early flowering during rice transformation (S6 Fig). The difference in flowering phenotype can also be explained by the reasons given for the mild flowering phenotype of *Arabidopsis* by *PaFT1* expression. In addition, the examination of the effect of phloem-specific *PaFT1* expression in *Arabidopsis* backgrounds where temperature-linked floral repressors are highly expressed showed that *PaFT1* at least partially overcomes the activity of the repressors such as FRI/FLC and SVP by up-regulating the expression of common target genes such as *SOC1* and *FUL* for floral induction indicating molecular functional conservation of PaFT1 as a floral activator in *Arabidopsis*. Complementation of *ft* mutants by the *PaFT1* genomic clone also suggests that the promoter and coding region of *PaFT1* contribute to floral promotion in *Arabidopsis*. Furthermore, the GUS expression pattern of p*PaFT1*:*GUS* in *Arabidopsis*, except in the shoot apex, was reminiscent of *Arabidopsis FT* expression raising the possibility that parts of the promoter are also recognized by *trans*-acting factors acting on *cis*-regulatory regions of *Arabidopsis FT*. However, distinct expression patterns of *PaFT1* by low temperature and photoperiod in the orchid also suggest that ‘orchid-specific’ or ‘*P*. *aphrodite* subsp. *formosana*-specific’ *trans*-acting factors may control *PaFT1* expression under certain conditions. In rice, for instance, Ehd1 is a strong regulator of the expression of *Hd3a*, a rice *FT*, but has no homologue in the *Arabidopsis* genome and its upstream regulators such as Ehd2 and Ehd4 are also known to be ‘monocot-specific’ or ‘*Oryza*-genus-specific’ [77–79]. Inducible expression systems other than chemical sprays are likely to be useful in orchid flowering manipulation. We, therefore, tested heat-shock inducible expression of *PaFT1* in *Arabidopsis* and confirmed the activity of PaFT1 as a floral activator (S4 Fig).

*PaFD* that encodes a protein that interacts with PaFT1 was expressed in almost all the organs examined in the orchid. In particular, *PaFD* transcripts were relatively abundant in the developing inflorescences and the level of expression in leaves gradually increased with plant growth. Interestingly, rice *FD* genes such as *OsFD1*, *OsFD2* and *OsFD3* are also expressed in the leaves and stems and the interaction between Hd3a and OsFD1 is mediated by 14-3-3 proteins [10, 71]. PaFD was able to interact with other FT proteins from several plant species including *Arabidopsis*, rice and *Oncidium* orchid. Of note, the mutant form of PaFT1, PaFT1Y86H could also interact with PaFD indicating that a putative PaTFL1 may compete with PaFT1 for the interaction with PaFD as is the case in *Arabidopsis* [80]. Thus, the regulation of the FT and FD genes for floral induction is likely to be conserved in the orchid although the expression pattern of *PaFD* is different from that of *AtFD* which is shoot apex-specific in *Arabidopsis*. PaFD belongs to the ‘eudicot and non-*Poaceae* FD group’ together with AtFD [71]. Closer analysis of the FD protein sequence indicates that PaFD has a TSSAPF motif at its carboxyl end and most members of the group have a conserved T(S)SS(T)APF motif at the same position. Abe et al [68] showed the threonine (Thr) residue at position 282 plays a critical role in the interaction of AtFD with AtFT and the interaction is also mediated by 14-3-3 proteins through the phosphorylation of AtFD at the Thr 282 [81]. Our analyses of the interaction between PaFT1 and PaFD mutant forms showed the serine (Ser) residue at position 227 of PaFD, the positional equivalent of Thr 282 of AtFD is important for the PaFT1-PaFD interaction. Meanwhile, OsFD1 and the members of the ‘*Poaceae* FD1 group’ have a conserved VL(MP)SAPF motif at their carboxyl ends (S9 Fig). Since the site is similar to the recognition site of the 14-3-3 proteins, the functional difference among various FD proteins through the interactions with other proteins including FT-like proteins may be due to the two amino acids in front of the S(T)AP motif at the carboxyl terminals. For example, both serine and threonine in the T(S)SS(T)APF motif of FDs in the eudicot and non-*Poaceae* FD group can be phosphorylated and this post-translational modification may cause functional diversity through multiple phosphorylations at the14-3-3 recognition site. Partial complementation of the *Arabidopsis fd* mutant using p*AtFD*:*PaFD* construct implies that *PaFD* is able to replace the functional activity of *Arabidopsis FD* in floral promotion. Furthermore, transgenic rice plants expressing *PaFD* exhibited early heading with increased expression of two rice *AP1* homologues, *OsMADS14* and *OsMADS15*, which is distinct from the results with rice *FD* overexpressors. Interestingly, neither rice *FD1* (*OsFD1*) nor *FD2* (*OsFD2*) overexpressors cause early heading although the proteins interact with Hd3a through 14-3-3 proteins [10, 71]. Since PaFD belongs to the ‘eudicot and non-*Poaceae* monocot FD group’ which is distinct from the *Poaceae* FD groups, the different activity or diverged function of the FDs may contribute to rice development at various steps (S9 Fig). Indeed, a recent report showed that *OsFD2* controls rice leaf development [71]. Although VIGS of *PaFD* was also applied to the orchid, we did not observe plants showing significantly delayed spiking with reduced expression level of *PaFD* (data not shown). In the case of *PaFT1*, however, more than 80% of the orchids treated with VIGS of *PaFT1* showed significant delayed spiking under inductive temperature compared with the control and the endogenous *PaFT1* expression level was also reduced suggesting that *PaFT1* at the very least plays a role in the initiation of florescence in the orchid. Recently, it was shown that increased level of *TaFT*, a *FT* from wheat could overcome the necessity of vernalization in flowering of wheat by a transgenic approach [82]. Thus, it will definitely be worthwhile examining whether transgenic orchids with increased level of *PaFT1* will initiate spiking under non-inductive high temperature.

## Conclusions

In conclusion, the present study demonstrates the potential roles of PaFT1 as a floral activator and its interacting protein PaFD in orchid flowering. Low ambient temperature is absolutely necessary, while the photoperiodic signal is not necessary for flowering of *P*. *aphrodite* subsp. *formosana*. The level of *PaFT1* expression correlates with the inductive environmental conditions and the flowering phenotype of *P*. *aphrodite* subsp. *formosana*. *PaFD* that encodes a PaFT1-interacting protein also shows flower-promoting activity in both *Arabidopsis* and rice. We, therefore, suggest the possibility that regulation of *FT* and *FD* genes in plants may have evolved and integrated into the distinct flowering circuits of plants to promote flowering under conditions favorable to each plant species. These findings broaden our understanding of various flowering processes of plants and provide potential tools for molecular breeding of orchid.

## Supporting Information

S1 Fig A detailed phylogenetic tree of FT proteins from various plants and proteins used for the construction of the tree. A, Phylogenetic tree of the deduced amino acid sequences of PaFT1 and FT sequences of other plant species. The tree was created with MEGA 5.2 using the neighbor-joining method and clustalW [83]. The accession numbers of the sequences are as follows: *Arabidopsis thaliana* (FT, BAA77838; TSF, BAA77840), *Citrus unshiu* (CiFT, AB027456), *Cymbidium goeringii* (CgFT, ADI58462), *Carica papaya* (CpFT, ACX85427), *Hordeum vulgare* (HvFT1, ABJ97441), *Ipomoea nil* (PnFT1, ABW73562), *Lactuca sativa* (LsFT, BAK14369), *Malus* x *domestica* (MdFT1, AB161112), *Oncidium* Gower Ramsey (OnFT, ACC59806), *Oryza sativa* (Hd3a, BAB61030; RFT1, BAO03187), *Populus nigra* (PnFT2a, AB109804), *Solanum lycopersicum* (SP3D, AY186735), *Solanum tuberosum* (StFT, ADA77529), *Triticum aestivum* (TaFT, ACA25437) and *Vitis vinifera* (VvFT, ACZ26523). The numbers at nodes represent the bootstrap values (with 1000 replicates) and the scale bar displays branch length. FTs from orchids are in a dotted box. B, PaFT1 has two conserved key amino acids 86-Tyr (▼) and 141-Gln (▼) which are believed to be important residues in FT proteins that act as floral activators [57–59].
(PDF)

S2 Fig The effect of ambient temperature on the expression of three *SOC1* homologues from *P*. *aphrodite* subsp. *formosana*. HT; high temperature (28°C/25°C as day and night temperature under LDs), LT; low temperature (23°C/20°C). The same RNAs used for Fig 1F were utilized for the analyses of gene expression. Recently, transcriptomic analyses using petals and lips of *P*. *amabilis*, a species that is closely related to *P*. *aphrodite*, identified eight *SOC1* genes [84]. Although the number of *SOC1* homologues that exist in *P*. *aphrodite* has not yet been reported, the expression of three reported *SOC1* homologues, *PaSOC1-1* (PATC136427), *PaSOC1-2* (PATC150808) and *PaSOC1-3* (PATC 154491) [85] was examined during the temperature shift.
(PDF)

S3 Fig Expression level of *PaFT1*, *OsMADS14* and *OsMADS15* in transgenic rice plants expressing *PaFT1*. Control is a transgenic rice plant containing an empty vector.
(PDF)

S4 Fig Effect of *PaFT1* under a heat-inducible expression system in *Arabidopsis*. A, Heat treated transgenic *ft-10* containing p*HSP*18.2:*PaFT1* showed earlier flowering than untreated plants. Sixteen-day-old seedlings of the plants were heat treated (2 hours from ZT 14 to ZT 16 under LDs at 37°C) for 3 weeks. *PaFT1* transcripts only highly accumulated in plants with heat treatment (in the box). B, Flowering time of plants with and without heat treatment. Three independent homozygous lines (14 to 22 individuals for each line) were tested for flowering time measurement. C and D, Heat treated transgenic plants (Col WT background) containing p*HSP*18.2:*PaFT1* showed earlier flowering than untreated plants. Three weeks old seedlings of the plants grown under SD (10 h light) were heat treated (2 hours from ZT 8 to ZT 10 at 37°C) for 3 weeks. Two independent homozygous lines (14 and 17 individuals for each line) were tested for flowering time measurement. The asterisk indicates that heat-treated plants flowered earlier than untreated plants or control. P ≤ 0.005 (Student’s *t*-test).
(PDF)

S5 Fig Subcellular localization of FT, FD and FDP proteins and the interaction between them in plant cells. A and B in the left panel, CFP:AtFT in an *Arabidopsis* cell. C and D in the left panel, CFP:PaFT1 in an *Arabidopsis* cell. E and F in the left panel, YFP:AtFD in an *Arabidopsis* cell. G and H in the left panel, YFP:AtFDP in an *Arabidopsis* cell. A and B in the right panel, BiFC assay between YFPn:AtFT and YFPc:AtFD in an *Arabidopsis* cell. C and D in the right panel, BiFC assay between YFPn:PaFT1 and YFPc:AtFD in an *Arabidopsis* cell. E and F, BiFC assay between YFPn:PaFT1 and YFPc:AtFDP in an *Arabidopsis* cell. G, Measurement of florescence intensity in each BiFC assay. For the evaluation of the relative fluorescence intensities of nuclei in the BiFC experiments the hardware values of gain, offset and zoom on the Leica SP2 AOBS instrument were adjusted image the nuclei of the positive control (AtFT:cYFP + YFPn:AtFD) such that the values of the 8 bit color scale included 255 (brightest level). Imaging of the other BiFC pairs were under the the same hardware values.
(PDF)

S6 Fig Generation of transgenic rice plants overexpressing *Hd3a*. Transgenic rice plants containing p*Ubi*:*Hd3a* produce flowers in the callus during transformation. Bars = 5 mm.
(PDF)

S7 Fig Expression of *PaFT1* and *PaFD* in the orchid leaves. Leaf numbers of orchids used in this study (upper) and the expression of *PaFT1* and *PaFD* in each leaf.
(PDF)

S8 Fig Expression of *Arabidopsis AP1* and *SOC1* genes in p*FD*:*PaFD fd-3* plants compared with WT and *fd-3* mutant plants. Nine-day-old seedlings grown under LDs were used for RNA extraction.
(PDF)

S9 Fig PaFD and FD proteins from various plant species. A, A phylogenetic tree (by MEGA5.2) [83]showing PaFD (*) belongs to eudicots and non-*Poaceae* FD group. B, Alignment of FD proteins used for the construction of the tree. The SAP motif [71] is marked with a dotted line.
(PDF)

S10 Fig Observation of orchids treated with VIGS of *PaFT1* over time. (PDF)

S1 Table Primers used in this study. (PDF)

S2 Table FD proteins used for the construction of the phylogenetic tree in S9 Fig
(PDF)
