# Supplementary material for: Functional Characterization of Phalaenopsis aphrodite Flowering Genes PaFT1 and PaFD
Source: PLoS One. 2015 Aug 28;10(8):e0134987. doi: 10.1371/journal.pone.0134987 (PMC4552788; doi:10.1371/journal.pone.0134987)
Supplement: S1 Fig — A, Phylogenetic tree of the deduced amino acid sequences of PaFT1 and FT sequences of other plant species. The tree was created with MEGA 5.2 using the neighbor-joining method and clustalW [83]. The accession numbers of the sequences are as follows: Arabidopsis thaliana (FT, BAA77838; TSF, BAA77840), Citrus unshiu (CiFT, AB027456), Cymbidium goeringii (CgFT, ADI58462), Carica papaya (CpFT, ACX85427), Hordeum vulgare (HvFT1, ABJ97441), Ipomoea nil (PnFT1, ABW73562), Lactuca sativa (LsFT, BAK14369), Malus x domestica (MdFT1, AB161112), Oncidium Gower Ramsey (OnFT, ACC59806), Oryza sativa (Hd3a, BAB61030; RFT1, BAO03187), Populus nigra (PnFT2a, AB109804), Solanum lycopersicum (SP3D, AY186735), Solanum tuberosum (StFT, ADA77529), Triticum aestivum (TaFT, ACA25437) and Vitis vinifera (VvFT, ACZ26523). The numbers at nodes represent the bootstrap values (with 1000 replicates) and the scale bar displays branch length. FTs from orchids are in a dotted box. B, PaFT1 has two conserved key amino acids 86-Tyr (▼) and 141-Gln (▼) which are believed to be important residues in FT proteins that act as floral activators [57–59]. (PDF) [file pone.0134987.s001.pdf]

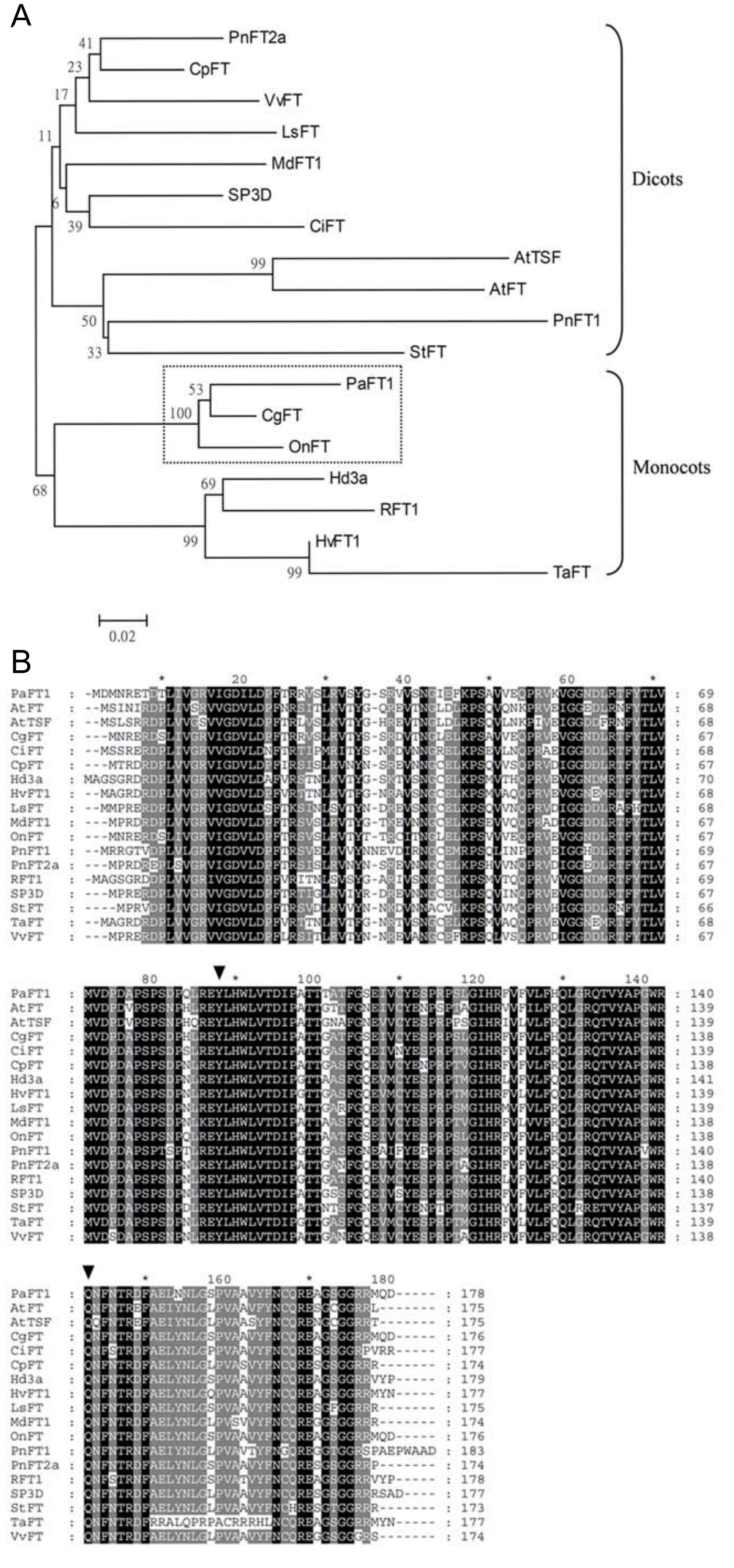

Figure S1. A detailed phylogenetic tree of FT proteins from various plants and proteins used for the construction of the tree.
