# Supplementary material for: Functional Characterization of Phalaenopsis aphrodite Flowering Genes PaFT1 and PaFD
Source: PLoS One. 2015 Aug 28;10(8):e0134987. doi: 10.1371/journal.pone.0134987 (PMC4552788; doi:10.1371/journal.pone.0134987)
Supplement: S2 Fig — HT; high temperature (28°C/25°C as day and night temperature under LDs), LT; low temperature (23°C/20°C). The same RNAs used for Fig 1F were utilized for the analyses of gene expression. Recently, transcriptomic analyses using petals and lips of P. amabilis, a species that is closely related to P. aphrodite, identified eight SOC1 genes [84]. Although the number of SOC1 homologues that exist in P. aphrodite has not yet been reported, the expression of three reported SOC1 homologues, PaSOC1-1 (PATC136427), PaSOC1-2 (PATC150808) and PaSOC1-3 (PATC 154491) [85] was examined during the temperature shift. (PDF) [file pone.0134987.s002.pdf]

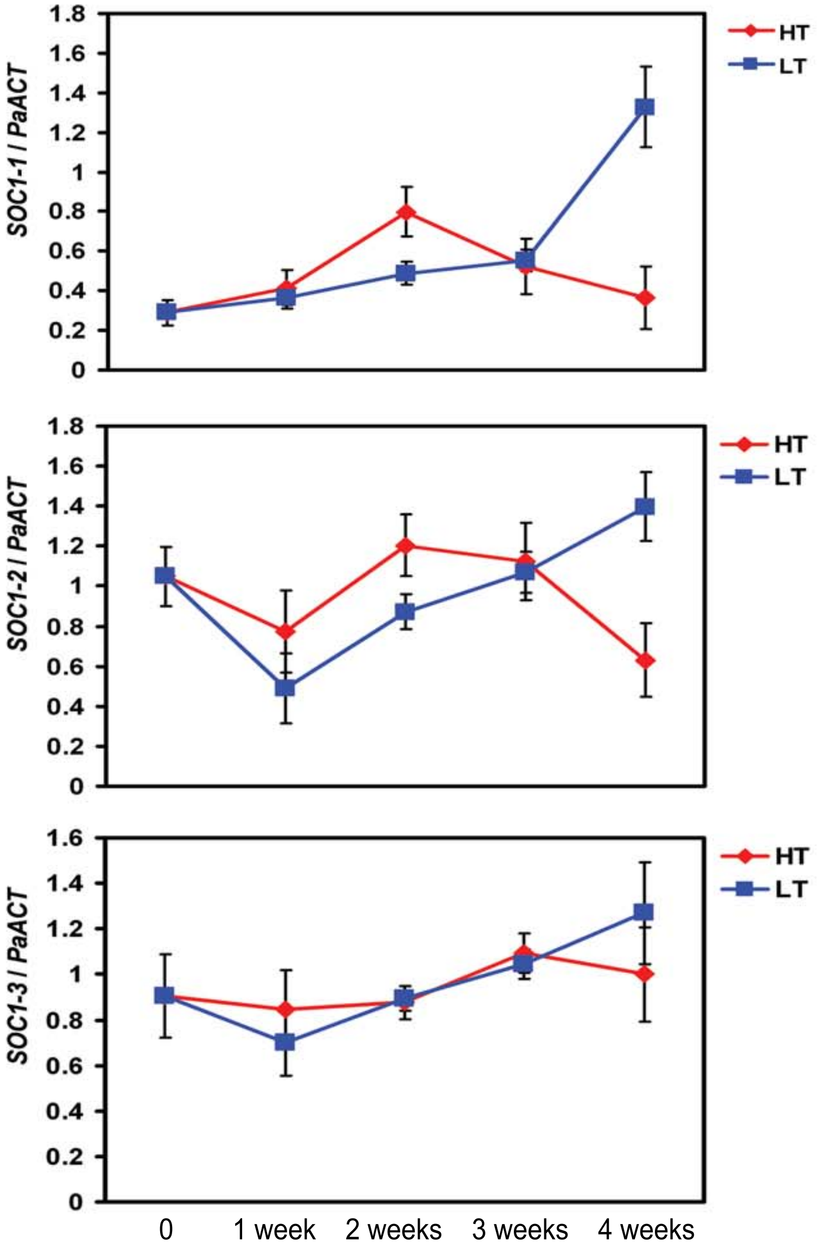

Figure S2. The effect of ambient temperature on the expression of three *SOC1* homologues from *P*. *aphrodite* subsp. *formosana*.
