# Supplementary figures and images for: Functional Characterization of Phalaenopsis aphrodite Flowering Genes PaFT1 and PaFD
Source: PLoS One. 2015 Aug 28;10(8):e0134987. doi: 10.1371/journal.pone.0134987 (PMC4552788; doi:10.1371/journal.pone.0134987)

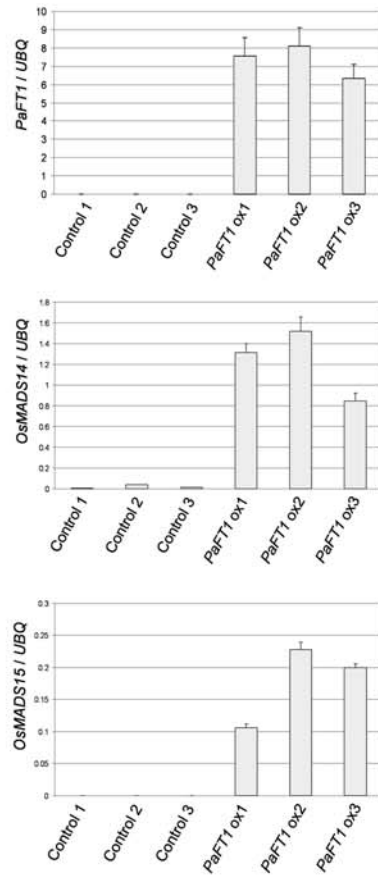

Figure S3. Expression level of *PaFT1*, *OsMADS14* and *OsMADS15* in transgenic rice plants expressing *PaFT1*.

Supplement: S3 Fig — Control is a transgenic rice plant containing an empty vector. (PDF) [file pone.0134987.s003.pdf]

A

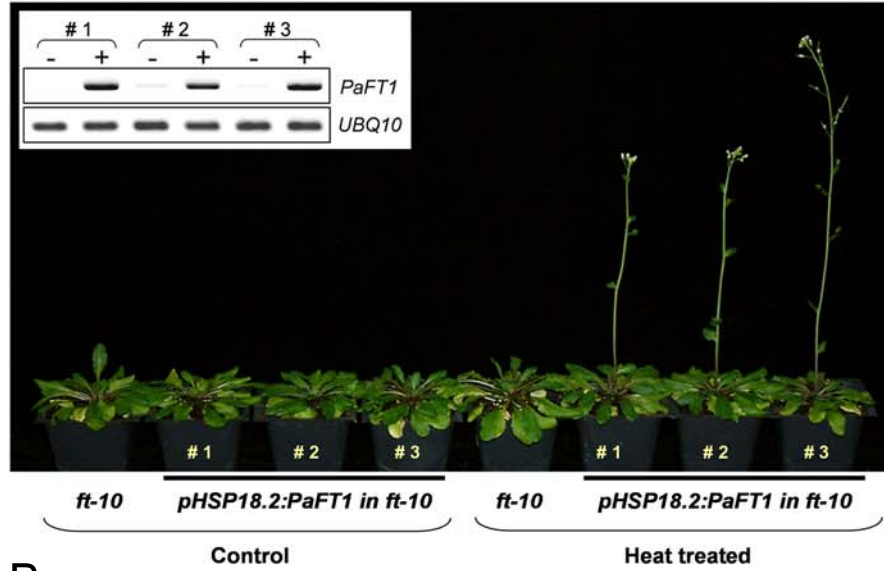

B

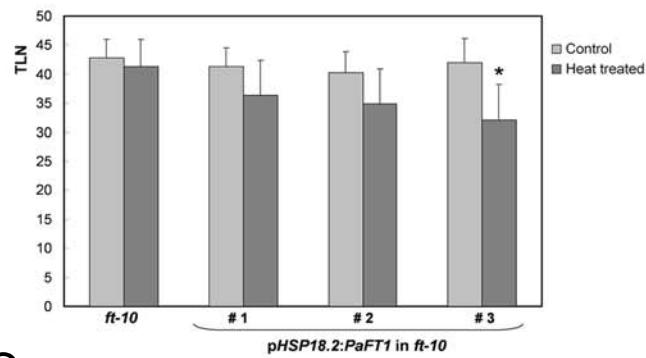

C

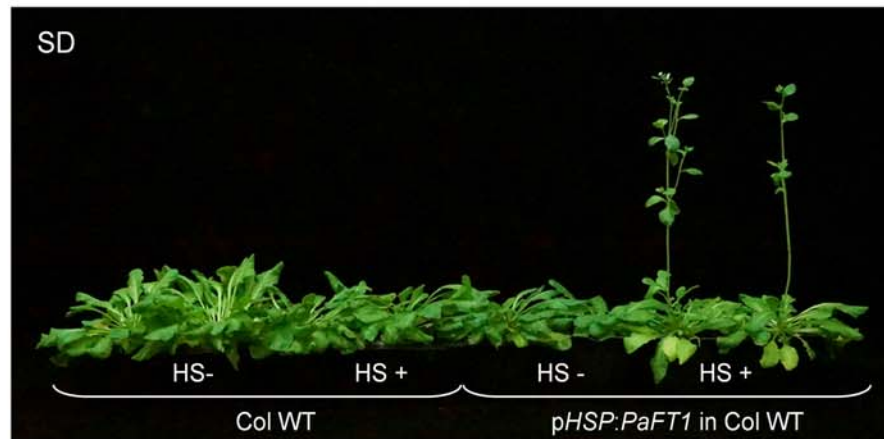

D

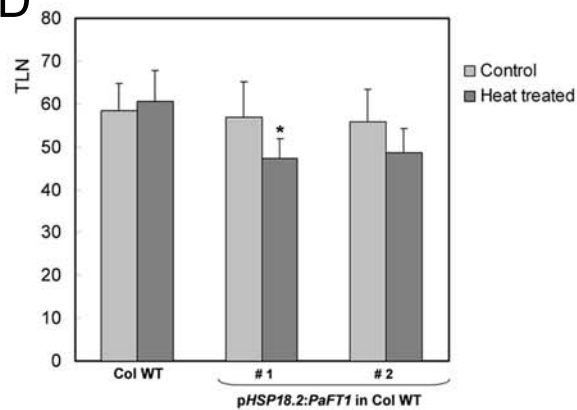

Figure S4. Effect of *PaFT1* under a heat-inducible expression system in Arabidopsis.

Supplement: S4 Fig — A, Heat treated transgenic ft-10 containing pHSP18.2:PaFT1 showed earlier flowering than untreated plants. Sixteen-day-old seedlings of the plants were heat treated (2 hours from ZT 14 to ZT 16 under LDs at 37°C) for 3 weeks. PaFT1 transcripts only highly accumulated in plants with heat treatment (in the box). B, Flowering time of plants with and without heat treatment. Three independent homozygous lines (14 to 22 individuals for each line) were tested for flowering time measurement. C and D, Heat treated transgenic plants (Col WT background) containing pHSP18.2:PaFT1 showed earlier flowering than untreated plants. Three weeks old seedlings of the plants grown under SD (10 h light) were heat treated (2 hours from ZT 8 to ZT 10 at 37°C) for 3 weeks. Two independent homozygous lines (14 and 17 individuals for each line) were tested for flowering time measurement. The asterisk indicates that heat-treated plants flowered earlier than untreated plants or control. P ≤ 0.005 (Student’s t-test). (PDF) [file pone.0134987.s004.pdf]

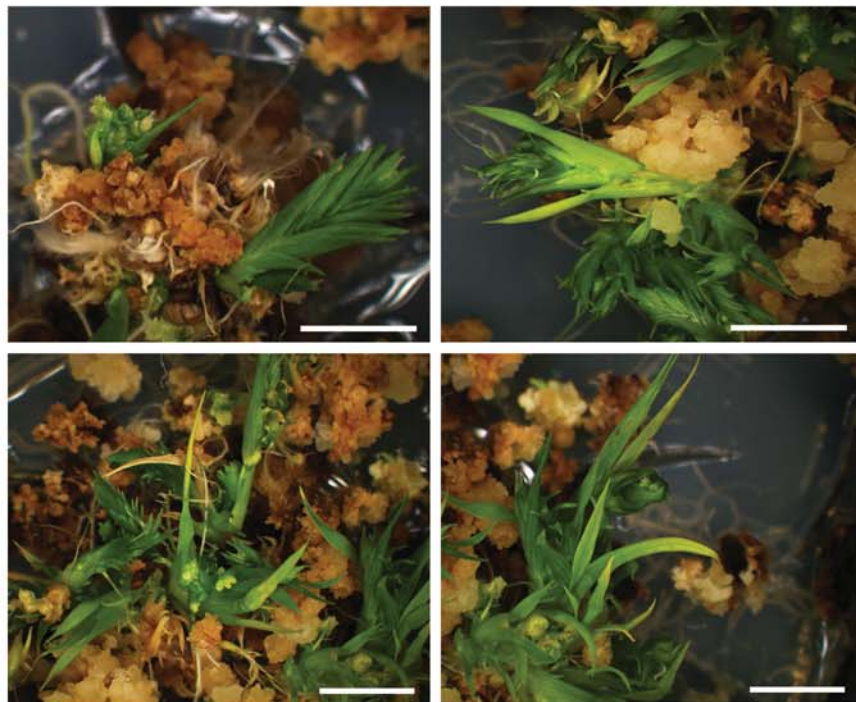

Figure S6. Generation of transgenic rice plants overexpressing *Hd3a*.

Supplement: S6 Fig — Transgenic rice plants containing pUbi:Hd3a produce flowers in the callus during transformation. Bars = 5 mm. (PDF) [file pone.0134987.s006.pdf]

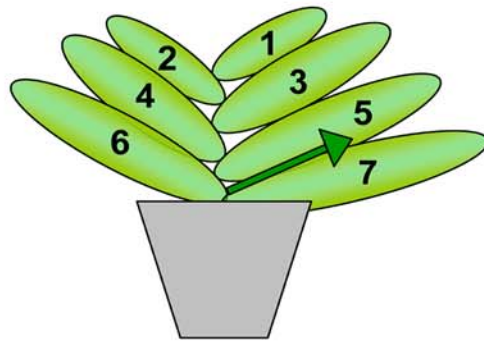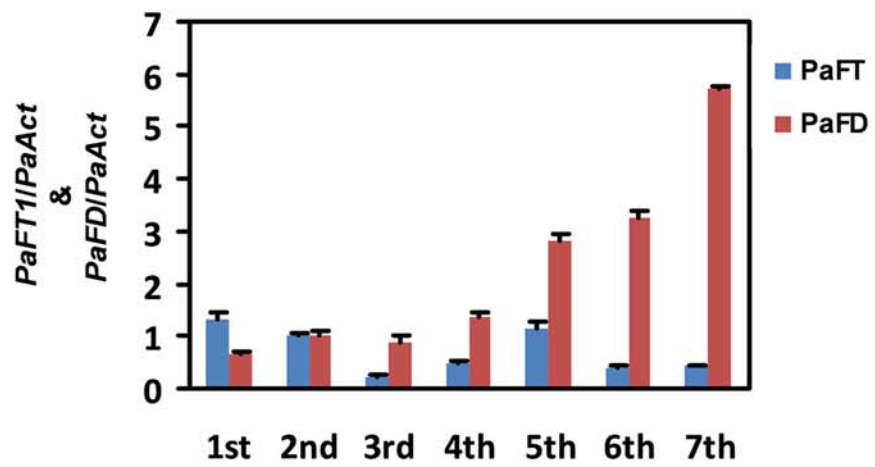

Figure S7. Expression of *PaFT1* and *PaFD* in the orchid leaves.

Supplement: S7 Fig — Leaf numbers of orchids used in this study (upper) and the expression of PaFT1 and PaFD in each leaf. (PDF) [file pone.0134987.s007.pdf]

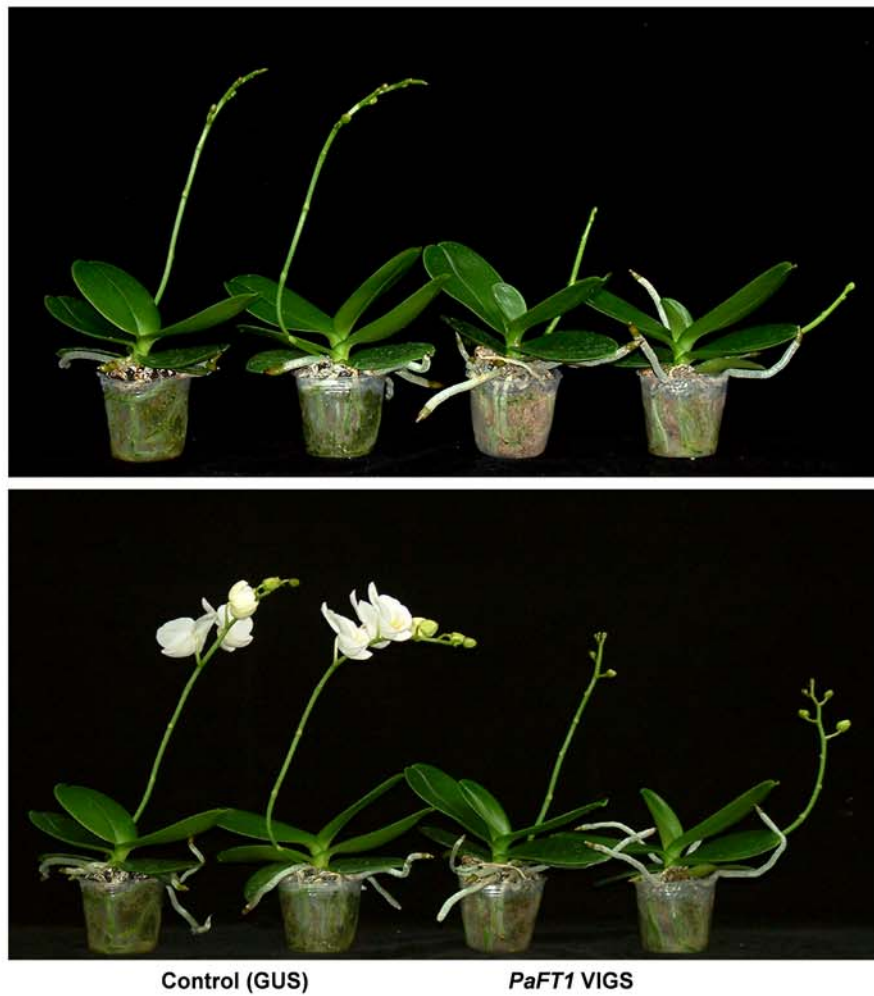

Figure S10. Observation of orchids treated with VIGS of *PaFT1* over time.

Supplement: S10 Fig — (PDF) [file pone.0134987.s010.pdf]
