# Supplementary material for: Functional Characterization of Phalaenopsis aphrodite Flowering Genes PaFT1 and PaFD
Source: PLoS One. 2015 Aug 28;10(8):e0134987. doi: 10.1371/journal.pone.0134987 (PMC4552788; doi:10.1371/journal.pone.0134987)
Supplement: S5 Fig — A and B in the left panel, CFP:AtFT in an Arabidopsis cell. C and D in the left panel, CFP:PaFT1 in an Arabidopsis cell. E and F in the left panel, YFP:AtFD in an Arabidopsis cell. G and H in the left panel, YFP:AtFDP in an Arabidopsis cell. A and B in the right panel, BiFC assay between YFPn:AtFT and YFPc:AtFD in an Arabidopsis cell. C and D in the right panel, BiFC assay between YFPn:PaFT1 and YFPc:AtFD in an Arabidopsis cell. E and F, BiFC assay between YFPn:PaFT1 and YFPc:AtFDP in an Arabidopsis cell. G, Measurement of florescence intensity in each BiFC assay. For the evaluation of the relative fluorescence intensities of nuclei in the BiFC experiments the hardware values of gain, offset and zoom on the Leica SP2 AOBS instrument were adjusted image the nuclei of the positive control (AtFT:cYFP + YFPn:AtFD) such that the values of the 8 bit color scale included 255 (brightest level). Imaging of the other BiFC pairs were under the the same hardware values. (PDF) [file pone.0134987.s005.pdf]

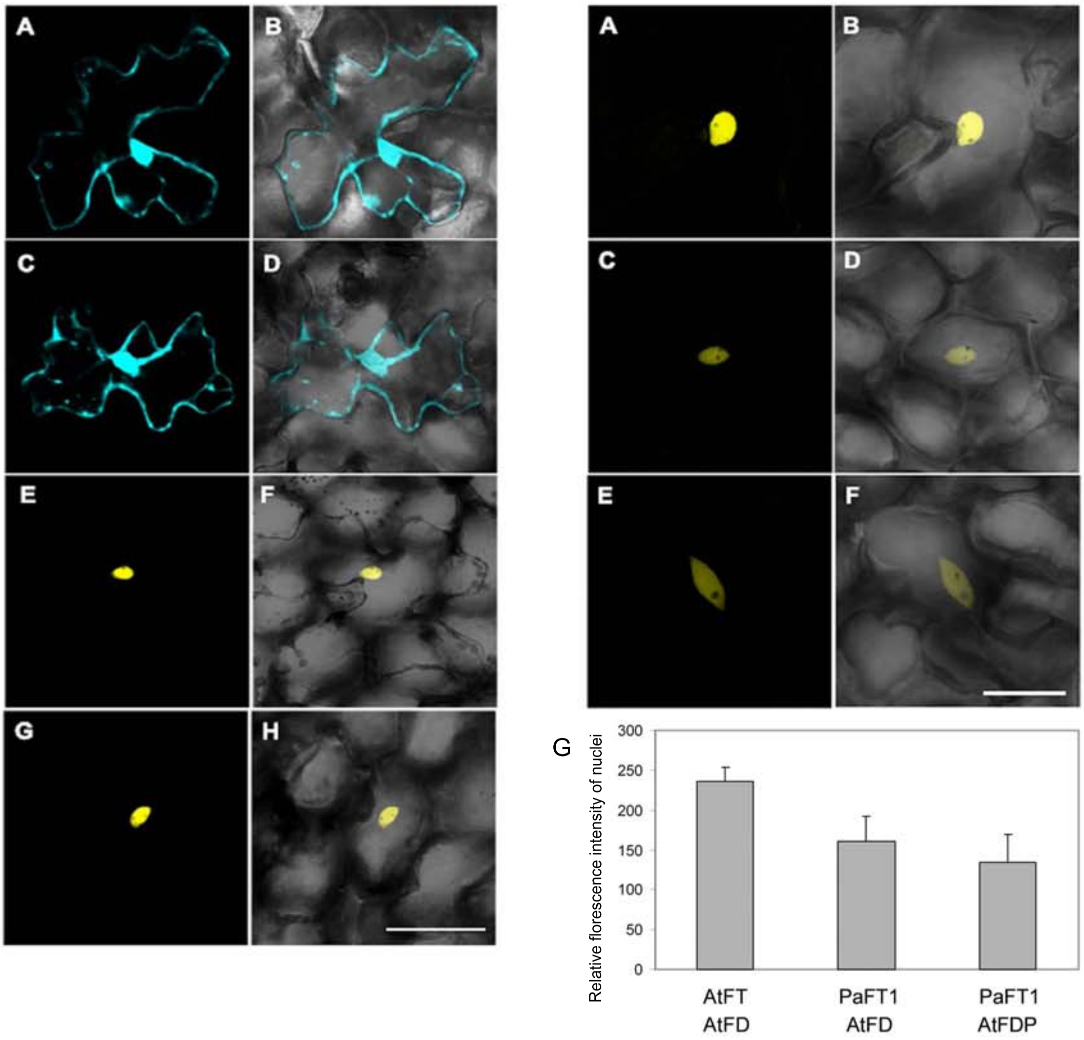

Figure S5. Subcellular localization of FT, FD and FDP proteins and the interaction between them in plant cells.
