# Supplementary material for: Functional Characterization of Phalaenopsis aphrodite Flowering Genes PaFT1 and PaFD
Source: PLoS One. 2015 Aug 28;10(8):e0134987. doi: 10.1371/journal.pone.0134987 (PMC4552788; doi:10.1371/journal.pone.0134987)
Supplement: S8 Fig — Nine-day-old seedlings grown under LDs were used for RNA extraction. (PDF) [file pone.0134987.s008.pdf]

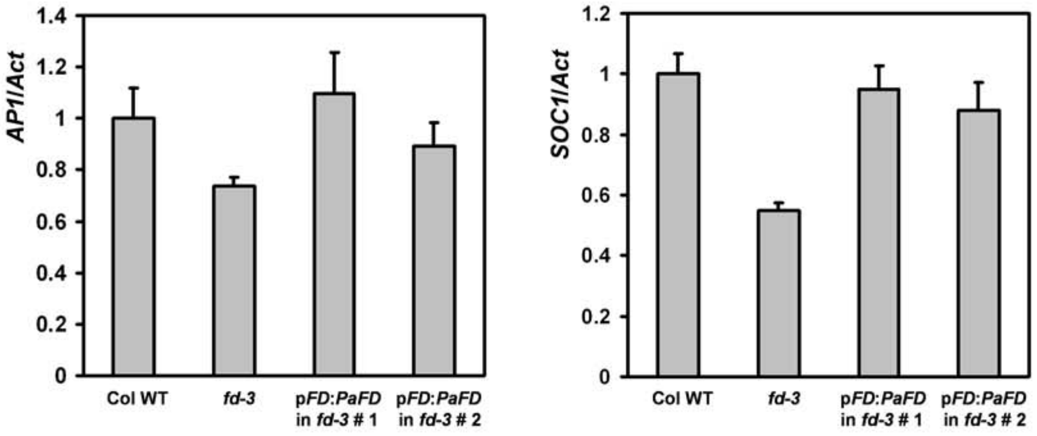

Figure S8. Expression of Arabidopsis *AP1* and *SOC1* genes in p*FD*:*PaFD fd-3* plants compared with WT and *fd-3* mutant plants.
