# Supplementary material for: Functional Characterization of Phalaenopsis aphrodite Flowering Genes PaFT1 and PaFD
Source: PLoS One. 2015 Aug 28;10(8):e0134987. doi: 10.1371/journal.pone.0134987 (PMC4552788; doi:10.1371/journal.pone.0134987)
Supplement: S1 Table — (PDF) [file pone.0134987.s011.pdf]

Table S1. Primers used in this study.

Primers	Sequence	Purpose
JIT267	5' TCCGACATAGAAGACATTATAGC 3'	PaFT1
JIT268	5' CAACATTCGGCAGTGAAATAGTG 3'	qPCR
JIT639	5' AGGCAATAGCTGCTCAGATTC 3'	PaFD
JIT640	5' TCTGGAGAGACAGATCTAC 3'	qPCR
JIT578	5' CTAGCGGAAACGCGACAGA 3'	PaACT
JIT579	5' CCAAGGGAAGCCAAAATGC 3'	qPCR
JIT258	5' CACTCTTGTCATGGTAGATCCAGATG 3'	PaFT1
JIT262	5' CAATCTTGCATCCTTCTTCCACCG 3'	RT-PCR
JIT619	5' ATGGAAGAAGTCTGGAAACACATTGAC 3'	PaFD
JIT620	5' TTAAAATGGCGCGGATGAAGTTCTCTG 3'	RT-PCR
JIT10F	5' GGCTAACAGAGAGAAGATGACC 3'	PaACT
JIT10R	5' AATAGACCCTCCAATCCAGAC 3'	RT-PCR
JIT588	5' GATCGCCAGTCGGTTGATTT 3'	PaUBQ
JIT589	5' GAAGCCTACGGCCATCGA 3'	qPCR
JIT1082	5' CCTGAGGTAGCCGTAGAAGCTC 3'	PaSOC1-1
JIT1083	5' TATGGAGATGTAACGGTCAC 3'	qPCR
JIT1077	5' GCAAAGTTAGATGGCGAAAGAAC 3'	PaSOC1-2
JIT1078	5' ACATTTGATTGTTATGCATGCCA 3'	qPCR
JIT1079	5' AGGAGATGGTTCCCCAATGTG 3'	PaSOC1-3
JIT1080	5' CGGGCCATCCTACATACAGTTC 3'	qPCR
JIT255	5' AGCTGCAGAAAACGAGAAGCTCTCTG 3'	AtSOC1
JIT256	5' GGGCTACTCTCTTCATCACCTCTTCC 3'	qPCR
JIT604	5′ TGCGTAACCTCCTCCAGAGATGGCTTTG 3′	AtFUL
JIT605	5′ GTTCTACTCGTTCGTAGTGGTAGGACGTAACATC 3′	qPCR
JIT515	5' ACAATATGCCTCCCCCTC 3'	AtAP1
JIT516	5' CTTCTTGATACAGACCACCC 3'	qPCR
JIT590	5' GGTAACATTGTGCTCAGTGGTGG 3'	AtActin
JIT591	5' AACGACCTTAATCTTCATGCTGC 3'	qPCR
JIT414	5' GATCTTTGCCGGAAAACAATTGGAGGATGGT 3'	AtUBQ10
JIT415	5' CGACTTGTCATTAGAAAGAAAGAGATAACAGG 3'	RT-PCR
JIT557	5' CAAGGACTTGACATTGAAGAGCTTCA 3'	AtSVP
JIT558	5' TGTCGGAGCTCTCGGAGTCAACAG 3'	RT-PCR
JIT562	5' GGGGACAAGTTTGTACAAAAAAGCAGGCTGCGTCCGTCCTGTAGAAACCCCAACCCGTGA 3'	GUS
JIT563	5' GGGGACCACTTTGTACAAGAAAGCTGGGTGTTCGGTATAAAGACTTCGCGCTGATACCAG 3'	VIGS
JIT427	5' GGGGACCACTTTGTACAAGAAAGCTGGGTACTCTCGGCTGCTCCACTACTGCCGAG 3'	PaFT1
JIT572	5' GGGGACAAGTTTGTACAAAAAAGCAGGCTGCATGGATATGAATAGAGAGACGGACAC 3'	VIGS
JIT641	5' GGGGACAAGTTTGTACAAAAAAGCAGGCTGCATGTGGCTCCTATCTCCTGCCGCCGATG 3'	PaFD
JIT827	5' GGGGACCACTTTGTACAAGAAAGCTGGGTGACTTCTTCCATACTTTTACGCTTTG 3'	VIGS
JIT600	5' TCCATCTTGGCATCTCTCAG 3'	OsACT
JIT601	5' GTACCCGCATCAGGCATCTG 3'	RT-PCR
JIT661	5' AACCAGCTGAGGCCCAAGA 3'	OsUBQ
JIT662	5' ACGATTGATTTAACCAGTCCATGA 3'	qPCR
JIT23	5' CGAGTGAAGATCCCTTTCTTGTTAC 3'	GUS
JIT7	5' CGCAAGACTGTAACCACGC 3'	RT-PCR
