# Supplementary material for: Functional Characterization of Phalaenopsis aphrodite Flowering Genes PaFT1 and PaFD
Source: PLoS One. 2015 Aug 28;10(8):e0134987. doi: 10.1371/journal.pone.0134987 (PMC4552788; doi:10.1371/journal.pone.0134987)
Supplement: S2 Table — (PDF) [file pone.0134987.s012.pdf]

Table S2. FD proteins used for the construction of the phylogenetic tree in Fig. S9.

**Species**		**Gene name**	**Locus ID/Accession No.**
FDs			
Rice	*Oryza sativa*	*OsFD1*	*Os09g0540800*
		OsFD2	*Os06g0720900*
		OsFD3	*Os02g0833600*
Maize	*Zea mays*	*DFL1*	*GRMZM2G067921*
Sorghum	*Sorghum bicolor*	*SbFD1*	*Sb02g031340*
Wheat	*Triticum aestivum*	*TaFD1*	*CK206464*
Barley	*Hordeum vulgare*	*HvFD1*	*BAK04622*
Banana	*Musa acuminata*	*MaFD1*	*GSMUA_Achr1T02630*
		MaFD2	*GSMUA_Achr5T11470_001*
		MaFD3	*GSMUA_Achr9G24090_001*
Date palm	*Phoenix dactylifera*	*PdFD1*	*PDK_30s1175071g003*
Arabidopsis	*Arabidopsis thaliana*	*AtFD*	*At4g35900*
		AtFDP	*At2g17770*
Tomato	*Solanum lycopersicum*	*SPGB*	*Solyc02g083520*
		SlFD2	*Solyc02g61990.2.1*
Potato	*Solanum tuberosum*	*StFD1*	*Sotub02g026810*
		StFD2	*Sotub02g009830*
Soybean	*Glycine max*	*GmFD1*	*Glyma04g02420*
		GmFD2	*Glyma06g02470*
Strawberry	*Fragaria vesca*	*FvFD1*	*mma14556.1-v1.0-hybrid*
Apple	*Malus × domestica Borkh.*	*MdFD1*	*MDP0000169473*
Poplar	*Populus trichocarpa*	*PtFD1*	*POPTR_0005s11140.1*
		PtFD2	*POPTR_0005s26480*
Grape	*Vitis vinifera*	*VvFD1*	*GSVIVT01009970001*
		VvFD2	*GSVIVT01006332001*
