# Supplementary material for: Functional Characterization of Phalaenopsis aphrodite Flowering Genes PaFT1 and PaFD
Source: PLoS One. 2015 Aug 28;10(8):e0134987. doi: 10.1371/journal.pone.0134987 (PMC4552788; doi:10.1371/journal.pone.0134987)
Supplement: S9 Fig — A, A phylogenetic tree (by MEGA5.2) [83]showing PaFD (*) belongs to eudicots and non-Poaceae FD group. B, Alignment of FD proteins used for the construction of the tree. The SAP motif [71] is marked with a dotted line. (PDF) [file pone.0134987.s009.pdf]

A

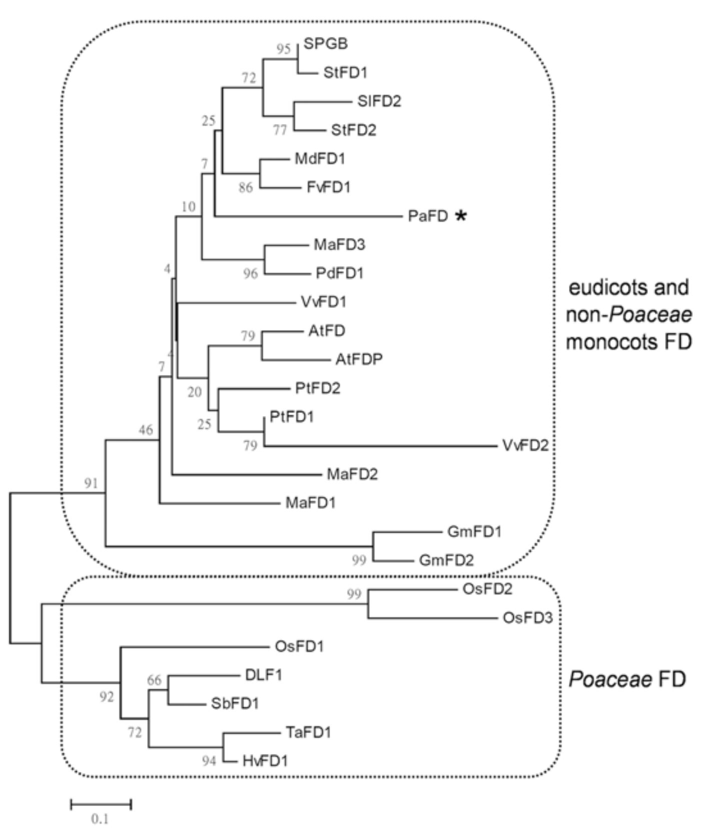

B

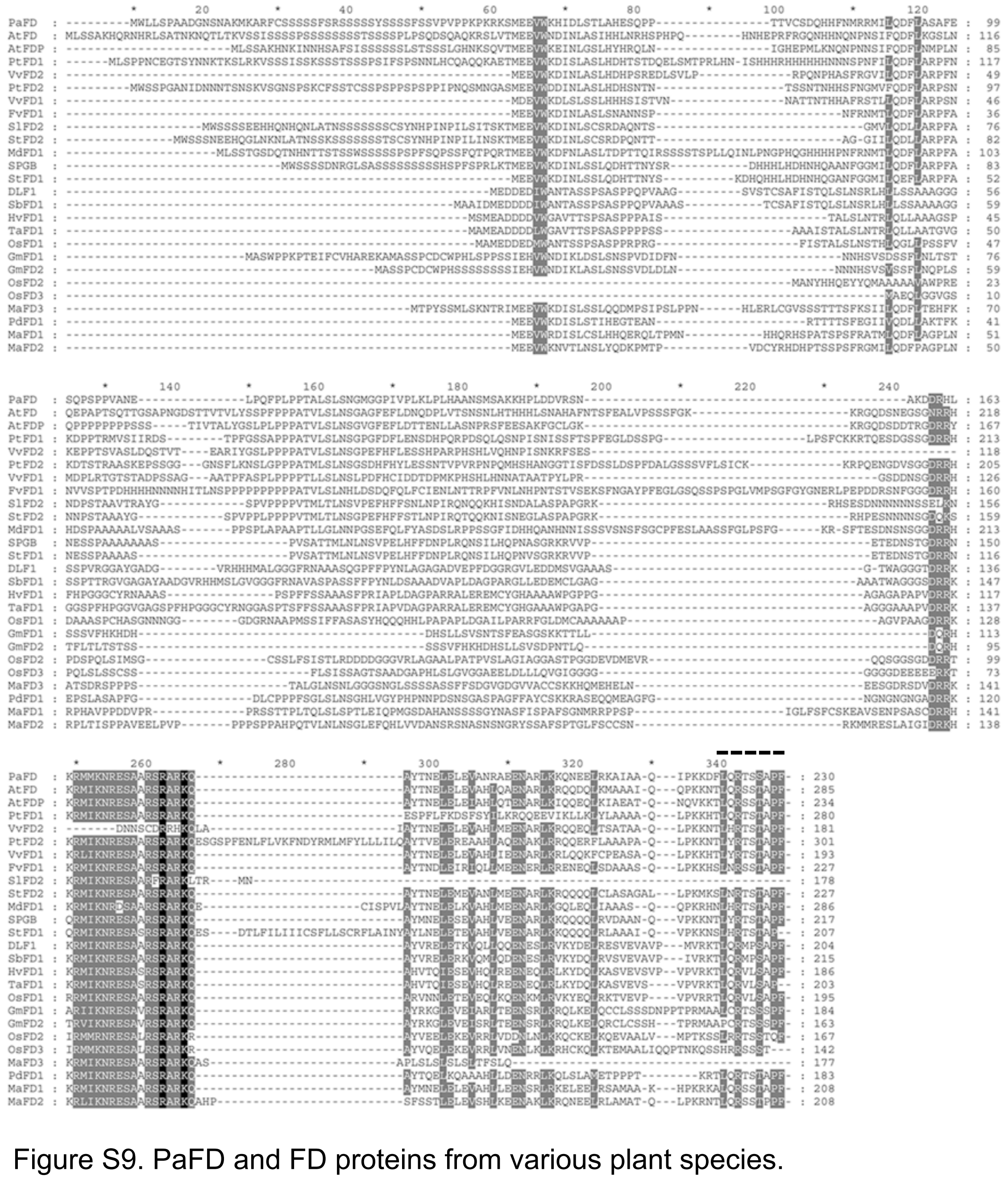

Figure S9. PaFD and FD proteins from various plant species.
